# Casimir-mediated amplification of flexural modes in suspended graphene

**DOI:** 10.1039/d6na00471g

**Published:** 2026-08-18

**Authors:** Sofia Ribeiro, Hugo Terças

**Affiliations:** a Max Planck Institute for the Science of Light Staudtstraße 2 D-91058 Erlangen Germany sofia.ribeiro@mpl.mpg.de; b Instituto Superior de Engenharia de Lisboa, Instituto Politécnico de Lisboa Rua Conselheiro Emídio Navarro 1 1959-007 Lisboa Portugal; c Instituto de Plasmas e Fusão Nuclear, Instituto Superior Técnico, Universidade de Lisboa Av. Rovisco Pais 1 1049-001 Lisboa Portugal hugo.tercas@isel.pt

## Abstract

We develop a minimal theoretical framework to describe near-field Casimir-mediated coupling between two suspended nanomembranes: a graphene sheet and a gold-coated GaAs membrane separated by a micrometer-scale vacuum gap. Coherent excitation of flexural (out-of-plane) modes in the gold membrane, provided by an external piezoelectric drive, enables efficient energy transfer to graphene through static vacuum electromagnetic coupling. Using a coupled-mode description equivalent to the classical equations of motion, we identify hybridized flexural modes exhibiting avoided crossings, tunable spectral response, and drive-dependent energy exchange. Under experimentally accessible conditions, Casimir-mediated mode hybridisation transfers energy from the driven gold membrane to graphene at rates comparable to or exceeding the intrinsic mechanical losses of graphene, resulting in a strong enhancement of its flexural response and linewidth narrowing. The Casimir interaction acts here solely as a static, non-contact coupling mechanism, while all energy is supplied externally through coherent mechanical driving, yielding drive-induced amplification without intrinsic negative damping. Our results establish Casimir coupling as a practical resource for controlling flexural dynamics in graphene nanomembranes and provide a platform for non-contact energy transfer and mode-selective amplification in two-dimensional mechanical systems.

## Abstract 2

Casimir-mediated coupling transfers energy from a piezo-driven Au/GaAs membrane to suspended graphene, producing hybridized flexural modes and strong amplification.

## Graphical abstract

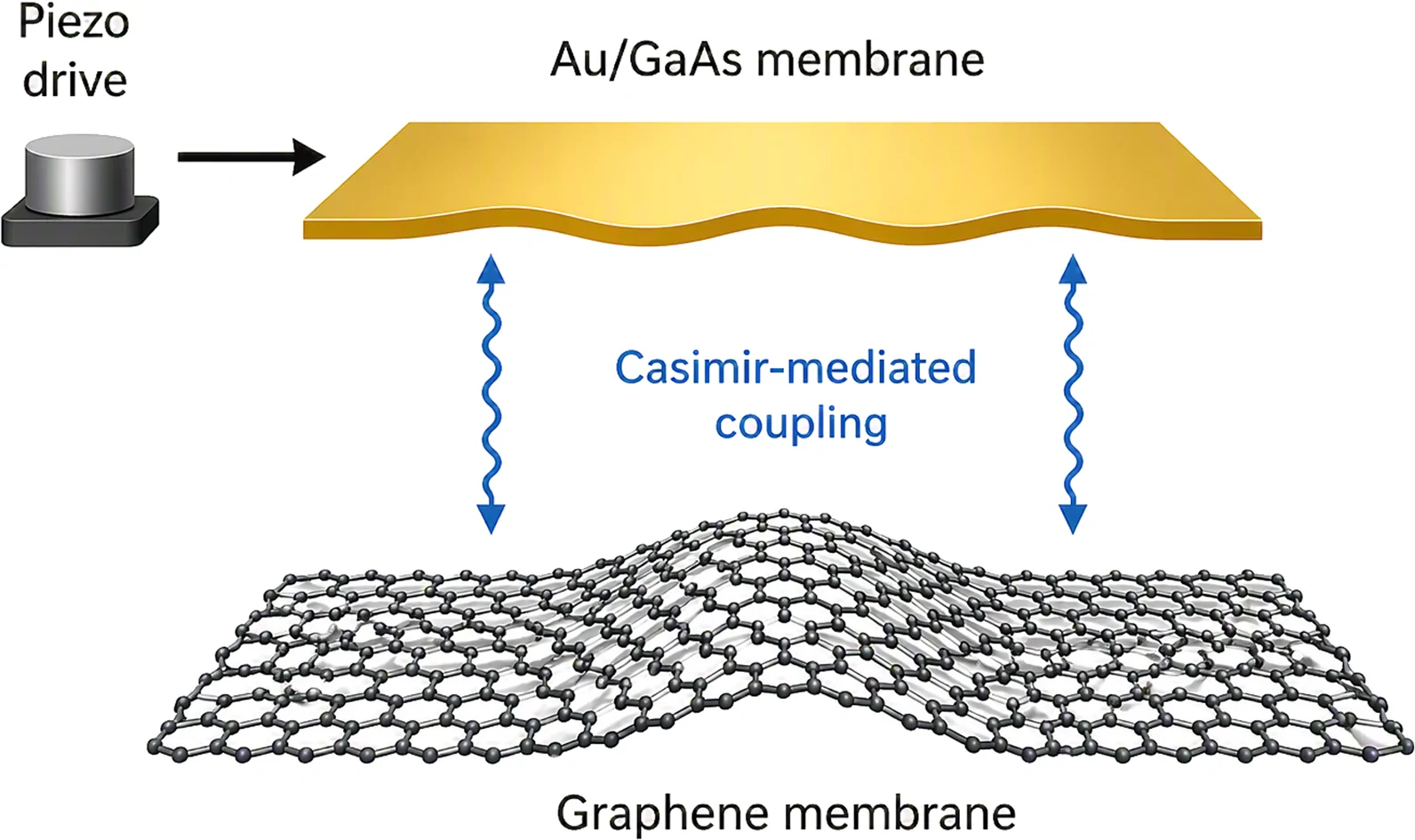

Out-of-plane vibrational modes are intrinsic excitations of solid plates. Their propagation in planar geometries was first analyzed by Rayleigh^1^ and later generalized by Lamb,^2^ giving rise to the well-known family of guided elastic waves, now referred to as Lamb modes. Lamb modes generalize flexural (out-of-plane) phonons for membranes with finite thickness and can be regarded as the continuum-mechanics analog of phonon modes in atomically thin membranes. Advances in micro- and nano-fabrication, particularly the isolation of two-dimensional (2D) materials such as graphene,^3,4^ have renewed interest in these modes. Owing to its atomic thickness and exceptional mechanical flexibility, single-layer graphene supports pronounced out-of-plane vibrations that strongly influence its thermal, mechanical, and electronic properties.^5–10^ Among these, the out-of-plane acoustic (ZA) flexural modes correspond to the lowest-frequency solutions and are readily excited, often dominating the vibrational spectrum due to their quadratic dispersion and low restoring force. In ideal, free-standing membranes at long wavelengths, ZA modes follow *ω*(*q*) ∝ *q*^2^, reflecting bending rigidity rather than tension. Substrates, built-in strain, or environmental interactions can modify this dispersion, introducing linear behavior or gaps.

At nanometer- to micrometer-scale separations, Casimir forces – arising from quantum vacuum fluctuations of the electromagnetic field^11^ – dominate the interaction between neutral bodies.^12,13^ Beyond static forces, Casimir interactions can dynamically couple mechanical degrees of freedom. Recent experiments show that Casimir-mediated interactions enable non-contact energy transfer between vibrating nanomembranes,^14^ suggesting new opportunities for controlling energy flow in nanoscale systems and for designing quantum thermal machines.^15^ While the static and thermal effects of Casimir interactions are well established,^16–18^ including in graphene systems,^19–24^ their role in the dynamical response of low-dimensional mechanical systems remains largely unexplored. In particular, the interplay between vacuum fluctuations and out-of-plane modes opens the possibility for Casimir-induced mechanical instabilities and amplification.^25–27^ This is especially relevant in atomically thin membranes, where flexural modes are highly sensitive to external forces,^5,28^ allowing parametric instabilities or resonant enhancements under controlled driving conditions.

In this work, we investigate conditions under which Casimir forces can amplify out-of-plane motion – flexural phonons, hereafter referred to as flexural modes – in suspended membranes. Focusing on the flexural spectrum, we analyze how membrane separation and material properties influence the dynamical stability of the system. The amplification mechanism we consider relies on Casimir-mediated coupling between a graphene sheet and a gold-coated GaAs membrane, both suspended over a trench and separated by a few micrometers (see Fig. 1), a geometry compatible with state-of-the-art suspended nanomembranes and heterogeneous nanomechanical platforms.^29^ Mechanical energy is supplied externally *via* a piezoelectric drive applied to the gold membrane, while Casimir interactions mediate vacuum-fluctuation-induced scattering between the hybridized flexural modes, enabling efficient, non-radiative energy transfer to the graphene membrane.

**Fig. 1:**
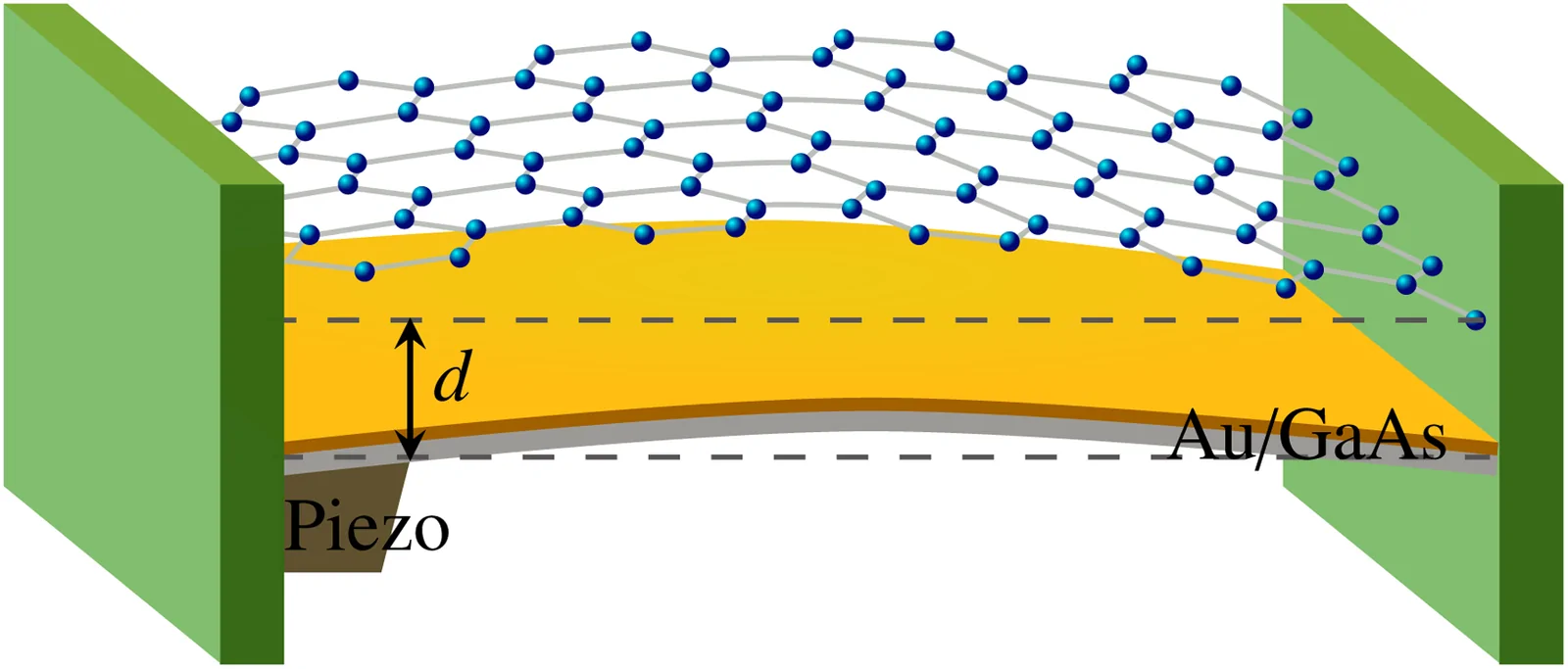
(Color online) Schematic of the proposed experimental setup (not to scale). Two nanomechanical resonators – one graphene and one Au/GaAs membrane – are separated at an average distance *d* and interact *via* Casimir forces. A piezoelectric actuator selectively drives the flexural (out-of-plane) mode of the Au/GaAs resonator, enabling indirect excitation of vibrations in the graphene membrane through vacuum fluctuation-mediated coupling.

Throughout the manuscript, we therefore use the term “Casimir-mediated parametric amplification” to emphasize that the Casimir interaction acts solely as a static, mode-selective coupling mechanism between flexural modes, while all energy is supplied by the external drive applied to the Au/GaAs membrane. The dynamics of interest are fully captured by a classical coupled-oscillator model; a harmonic-oscillator Hamiltonian formulation is introduced only as a compact representation of the same equations of motion and as a convenient basis for identifying hybridized normal modes and comparing with standard optomechanical models. In this sense, the system realizes a laser-like mechanical amplifier in a classical, driven-dissipative regime: under realistic experimental conditions, Casimir-mediated coupling can transfer energy from the driven gold membrane to the graphene membrane at a rate that compensates its intrinsic mechanical losses, leading to selective mode amplification and linewidth narrowing, analogous to gain narrowing in optical lasers but arising entirely from driven, classical parametric dynamics. These results provide a quantitative framework for Casimir-enabled mode transfer and amplification in two-dimensional mechanical systems, and identify experimentally accessible regimes for controlling flexural dynamics in graphene *via* near-field vacuum forces.

## I. Mode coupling mechanism

We consider the coupled dynamics of two suspended nanomembranes – a graphene sheet and a gold-coated GaAs membrane – separated by a micrometer-scale vacuum gap and interacting *via* Casimir forces (see Fig. 1). The central physical process addressed in this work is non-contact energy transfer from an externally driven nanomembrane to an undriven one through Casimir-mediated near-field vacuum electromagnetic coupling. In the parameter regime of interest, the relevant flexural modes exhibit large classical occupations, and their dynamics are accurately described by classical equations of motion.

For clarity, we begin with a classical description of the driven, coupled nanomechanical system. A harmonic-oscillator Hamiltonian formulation is subsequently introduced as a compact and equivalent representation of the same dynamics, providing a convenient basis for identifying hybridized normal modes and for direct comparison with standard coupled-mode descriptions used in nanomechanics and optomechanics. Throughout this section, the Casimir interaction acts solely as a static, mode-selective coupling mechanism; all energy injected into the system is supplied externally through coherent mechanical driving of the gold-coated membrane.

Restricting attention to a single flexural mode with fixed in-plane wavevector *k*, the system can be modeled as two damped nanomechanical oscillators coupled by an effective spring constant arising from the Casimir interaction. We write the canonical position and momentum variables (*X*_*c*_, *P*_*c*_) in terms of harmonic-oscillator amplitudes *c* ∈ {*a*, *b*} (gold and graphene modes, respectively) as1
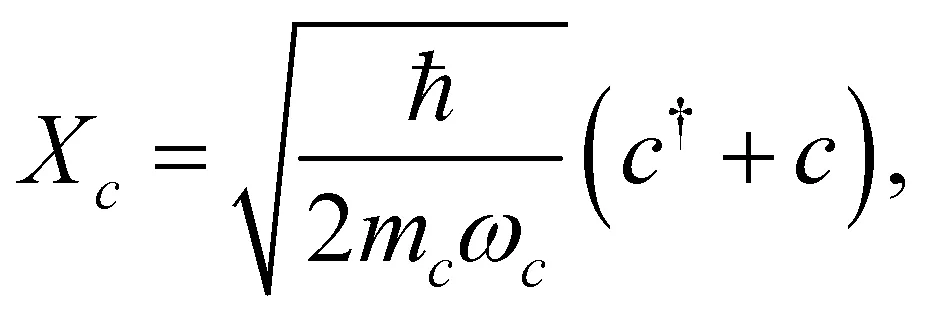
2
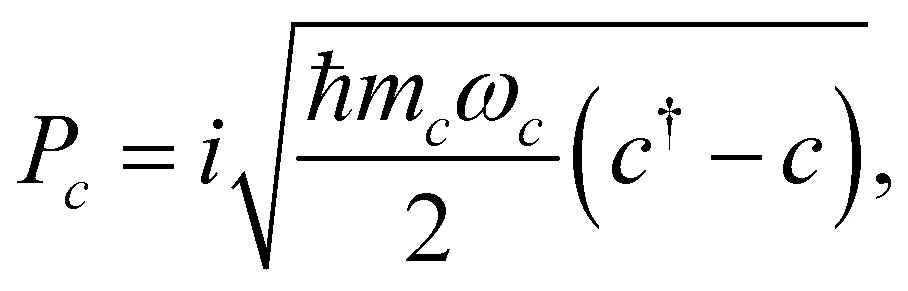
where *c* may be interpreted either as a quantum annihilation operator or, in the present classical context, as a complex mode amplitude.

Including an external coherent drive applied to the gold membrane, the total Hamiltonian reads3
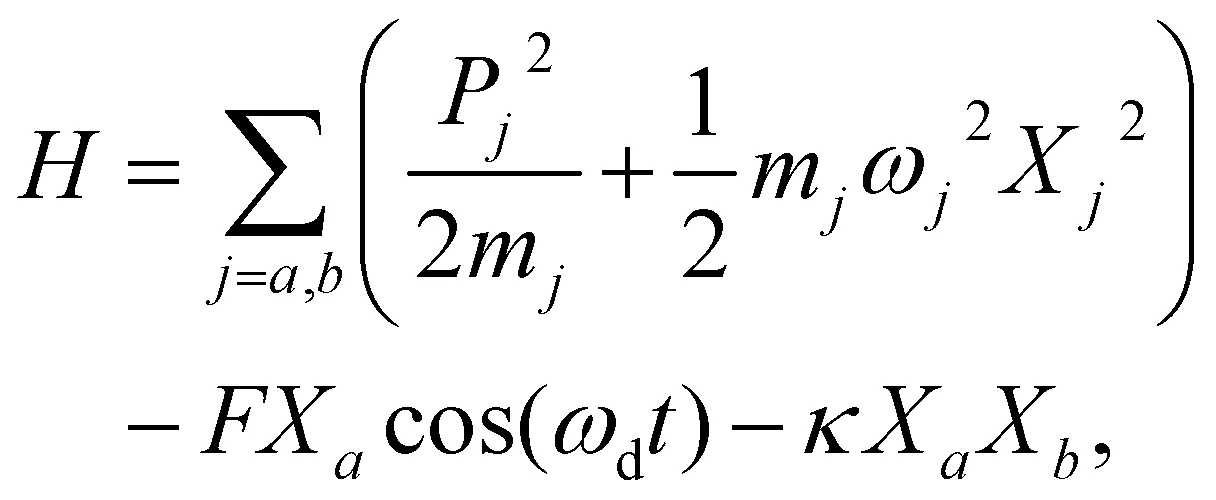
where *F* and *ω*_d_ denote the amplitude and frequency of the drive. The parameter 
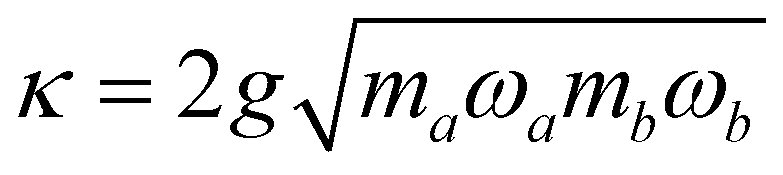
 encodes the Casimir-mediated coupling between the flexural modes, with the coupling strength *g* defined below.

The corresponding classical equations of motion are

4 *Ẍ*_*a*_ + *Γ*_*a*_*Ẋ*_*a*_ + *ω*_*a*_^2^*X*_*a*_ = *κ*_*a*_*X*_*b*_ + *Λ*_*a*_ cos(*ω*_d_*t*),

5 *Ẍ*_*b*_ + *Γ*_*b*_*Ẋ*_*b*_ + *ω*_*b*_^2^*X*_*b*_ = *κ*_*b*_*X*_*a*_,

with *κ*_*c*_ = *κ*/*m*_*c*_ and *Λ*_*a*_ = *F*/*m*_*a*_, and where *Γ*_*a*_ and *Γ*_*b*_ are the intrinsic mechanical damping rates of the gold and graphene membranes, respectively. These equations describe a driven, dissipative nanomechanical system in which energy injected into the gold membrane can be coherently transferred to graphene through Casimir-mediated coupling.

For the purpose of mode-hybridization analysis, it is convenient to work in a harmonic-oscillator representation in *k*-space and to omit the explicit driving term. Restricting again to a single flexural mode with fixed in-plane wavevector *k*, the coupled system can be written as6
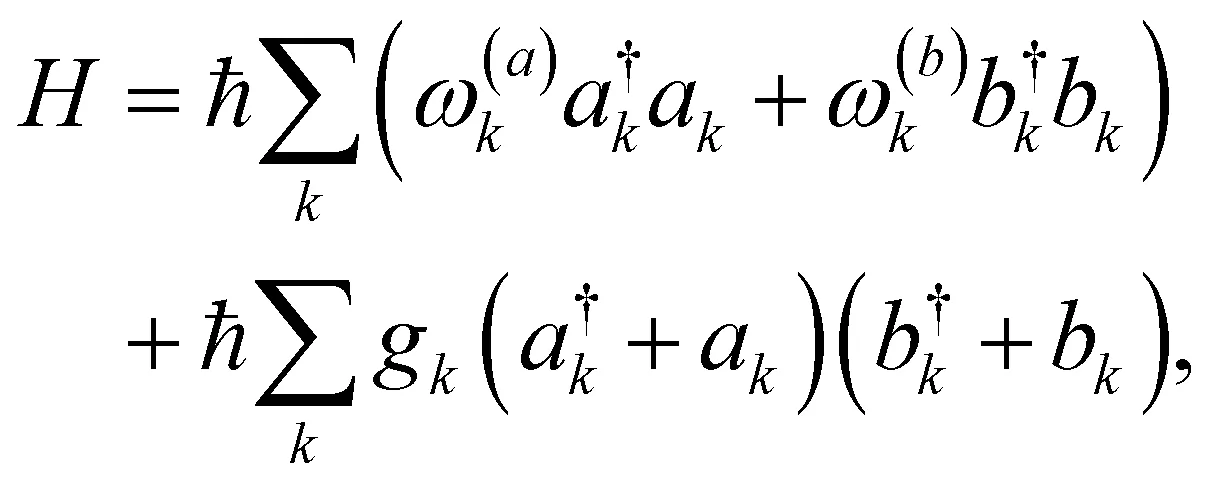
where *a*_*k*_ and *b*_*k*_ denote the amplitudes of the flexural modes of the gold and graphene membranes, respectively. When interpreted classically, these amplitudes correspond to complex mode envelopes, and the bilinear term represents an effective spring-like coupling consistent with eqn (4) and (5). The harmonic-oscillator formulation therefore serves as a concise notation for the same coupled nanomechanical dynamics and does not imply the generation of nonclassical states in the parameter regime considered here.

The Casimir-mediated coupling strength is given by^15^7
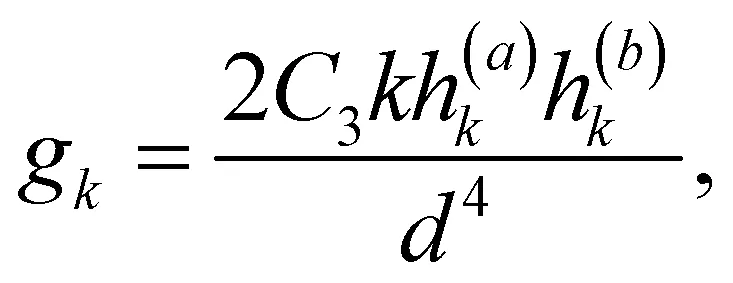
where *d* is the separation between the membranes, *C*_3_ is the material- and geometry-dependent Casimir coefficient, and the factor of 2 arises from summation over the polarization index. The quantities *h*^(*j*)^_*k*_ denote the zero-point displacement amplitudes of the flexural modes. Here *k* denotes the in-plane wavevector of the flexural deformation mode. Eqn (7) follows from the perturbative expansion of the Casimir energy between weakly deformed surfaces about an average separation *d*. It is valid for small displacement amplitudes compared with the mean separation, |*X*_*a*,*b*_| ≪ *d*, and for small surface slopes, *k*|*X*_*a*,*b*_| ≪ 1, so that higher-order deformation terms can be neglected. This form is closely related to the perturbative treatment of the Casimir interaction between corrugated or deformed plates developed in ref. 30 and 31.

Eqn (7) highlights the strong algebraic dependence of the coupling on the membrane separation, *g*_*k*_ ∝ *d*^−4^, characteristic of near-field Casimir interactions in planar nanostructures. While the Casimir pressure between parallel plates scales as *P*_Casimir_ ∝ *d*^−4^ and the local force gradient as *∂F*/*∂d* ∝ *d*^−5^,^12^ the effective mode–mode coupling relevant here arises from the expansion of the Casimir energy *E*(*d*) ∝ *d*^−3^ about the equilibrium separation. Linearization in the membrane displacements yields an effective spring-like interaction whose strength scales as *d*^−4^, as encoded in *g*_*k*_.^15^

For experimentally realistic parameters, a separation *d* = 1.5 µm yields Casimir-mediated coupling rates in the sub-megahertz regime, comparable to or exceeding the intrinsic mechanical damping rates of high-quality suspended graphene membranes at cryogenic temperatures (*e.g.*, *g* ∼ 2π × 0.2 MHz *versus Γ*_*b*_ ∼ 2π × 0.1 kHz). This regime enables efficient drive-induced energy transfer and amplification of graphene flexural motion when the gold membrane is coherently excited.

The same perturbative treatment of the Casimir energy that gives the intermembrane coupling also generates diagonal self-terms for each membrane. This point is already implicit in the proximity-force treatment of ref. 15. For a flexural ripple with in-plane wavevector *k*, the Casimir correction can be written schematically as8
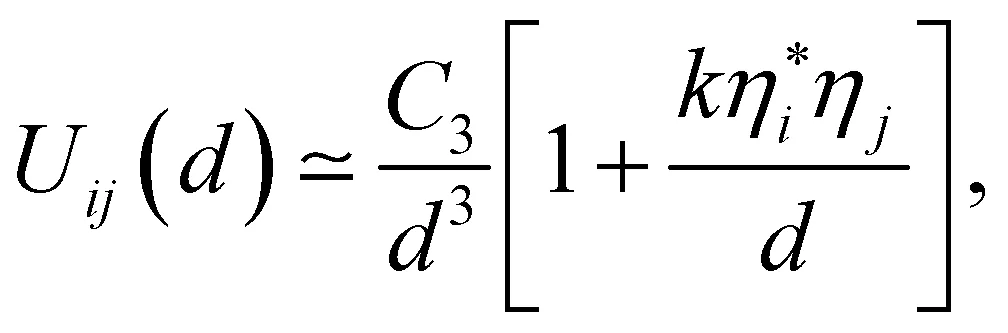
where *η*_*i*_ and *η*_*j*_ denote the out-of-plane displacement amplitudes of the two membranes. For *i* = *j*, this expression gives diagonal Casimir-induced self-energy corrections,9
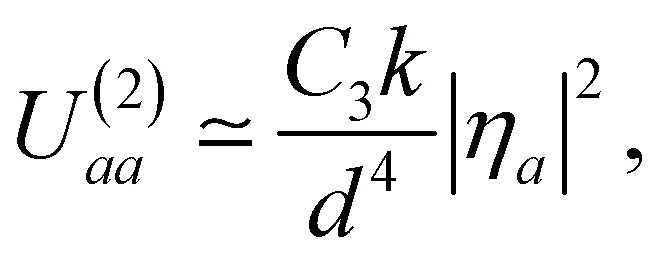
10
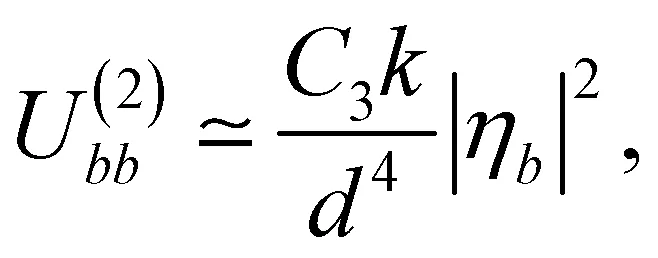
whereas for *i* ≠ *j* it gives the intermembrane coupling term11
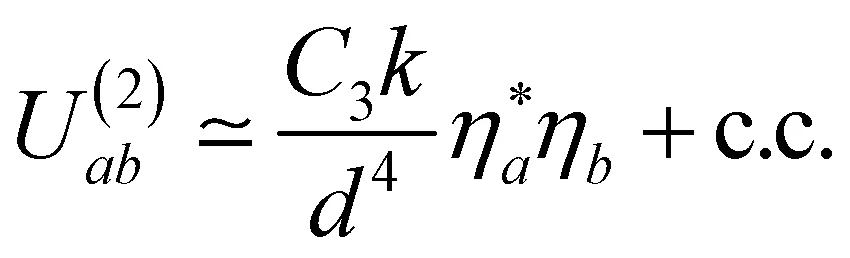


After quantization, the off-diagonal term yields the coupling rate12
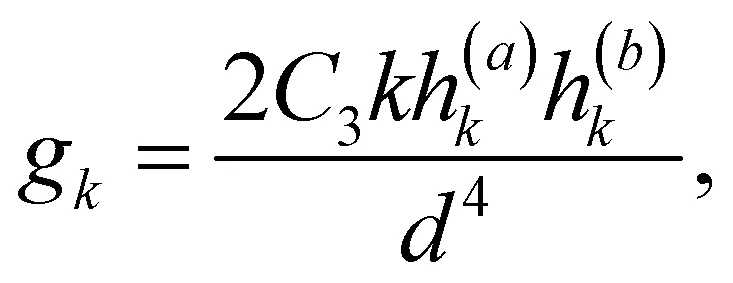
while the diagonal terms produce Casimir-induced frequency shifts,

13 *ω*^(*a*)^_*k*_ → *ω̃*^(*a*)^_*k*_ = *ω*^(*a*)^_*k*_ + *δω*^(*a*)^_*k*_,

14 *ω*^(*b*)^_*k*_ → *ω̃*^(*b*)^_*k*_ = *ω*^(*b*)^_*k*_ + *δω*^(*b*)^_*k*_.

In the notation of ref. 15, these shifts are of order15
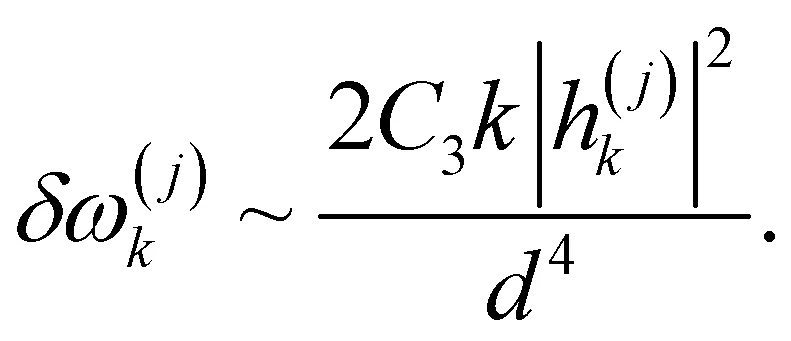


Thus, the diagonal self-terms and the off-diagonal coupling are generated at the same order in the Casimir expansion. In the present work, the diagonal terms are absorbed into the experimentally relevant, separation-dependent mechanical frequencies *ω*^(*a*)^_*k*_ and *ω*^(*b*)^_*k*_, whereas the off-diagonal term is retained explicitly because it is responsible for the avoided crossing, mode hybridization, and coherent energy transfer between the two membranes.

For notational simplicity, we drop the tilde in what follows and denote the renormalized frequencies by *ω*^(*a*)^_*k*_ and *ω*^(*b*)^_*k*_. We emphasize that these diagonal Casimir-induced self-terms are conservative in the static equilibrium regime considered here. They modify the real part of the mechanical susceptibility and therefore shift the resonance frequencies, but they do not by themselves generate negative damping. The damping modification discussed below arises from the dynamical coupling between the two membranes and, in the non-equilibrium case, from the additional pumping contribution *Γ*_NEQ_. Thus, the diagonal self-terms affect the location of the resonance and the avoided crossing, but they do not change the physical origin of the gain mechanism.

Diagonalization of the bilinear Hamiltonian in eqn (6) yields two hybridized normal modes corresponding to coherent superpositions of flexural motion in the two membranes. These modes exhibit avoided crossings as a function of in-plane wavevector, providing a clear spectral signature of Casimir-mediated coupling in nanomechanical systems, and the corresponding upper (U) and lower (L) hybridized frequencies are16
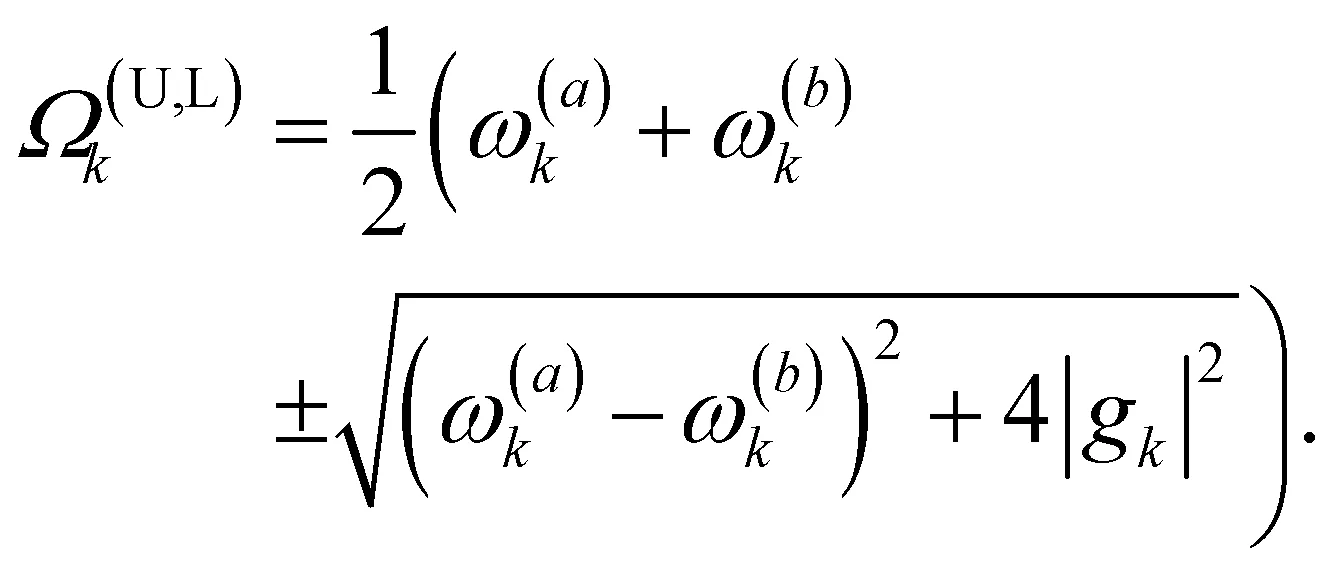


As discussed in the following sections, external driving of the gold membrane enables selective amplification of the graphene component of these hybridized modes, while the intrinsic damping remains positive in equilibrium.

## II. Spectral signatures of mode hybridization

The parameters considered here are compatible with current nanomechanical platforms. GaAs nanomembranes exhibit intrinsic quality factors of *Q* ∼ 10^6^, which decrease to *Q* ∼ 10^4^–2 × 10^4^ when coated with a gold film.^32^ Such suspended semiconductor nanomembranes have been extensively developed as versatile mechanical elements with low dissipation and excellent compatibility with external actuation and readout schemes, including piezoelectric and optical techniques, as demonstrated in several nanoscale studies.^29,33^

Suspended graphene nanoresonators can achieve even higher quality factors of up to *Q* ∼ 10^5^,^34^ and their flexural dynamics, tunability, and readout have been widely characterized in nanomechanical devices reported in *Nanoscale*, spanning regimes from MHz to GHz frequencies.^35^ The Casimir interaction in the present geometry is primarily governed by the gold–graphene interface,^36^ ensuring near-field coupling over the full membrane area, a configuration compatible with heterogeneous nanomembrane assemblies explored in previous nanoscale platforms.^33^

Piezoelectric actuation in the GaAs substrate enables dynamic excitation and tuning of the membrane resonance *via* voltage-induced stress,^37^ while graphene motion can be monitored using interferometric or optical techniques routinely employed in suspended nanomembrane experiments.^35^ Together, these elements place the proposed system well within experimentally established nanomechanical architectures.

### A. Polariton dispersion

**Fig. 2:**
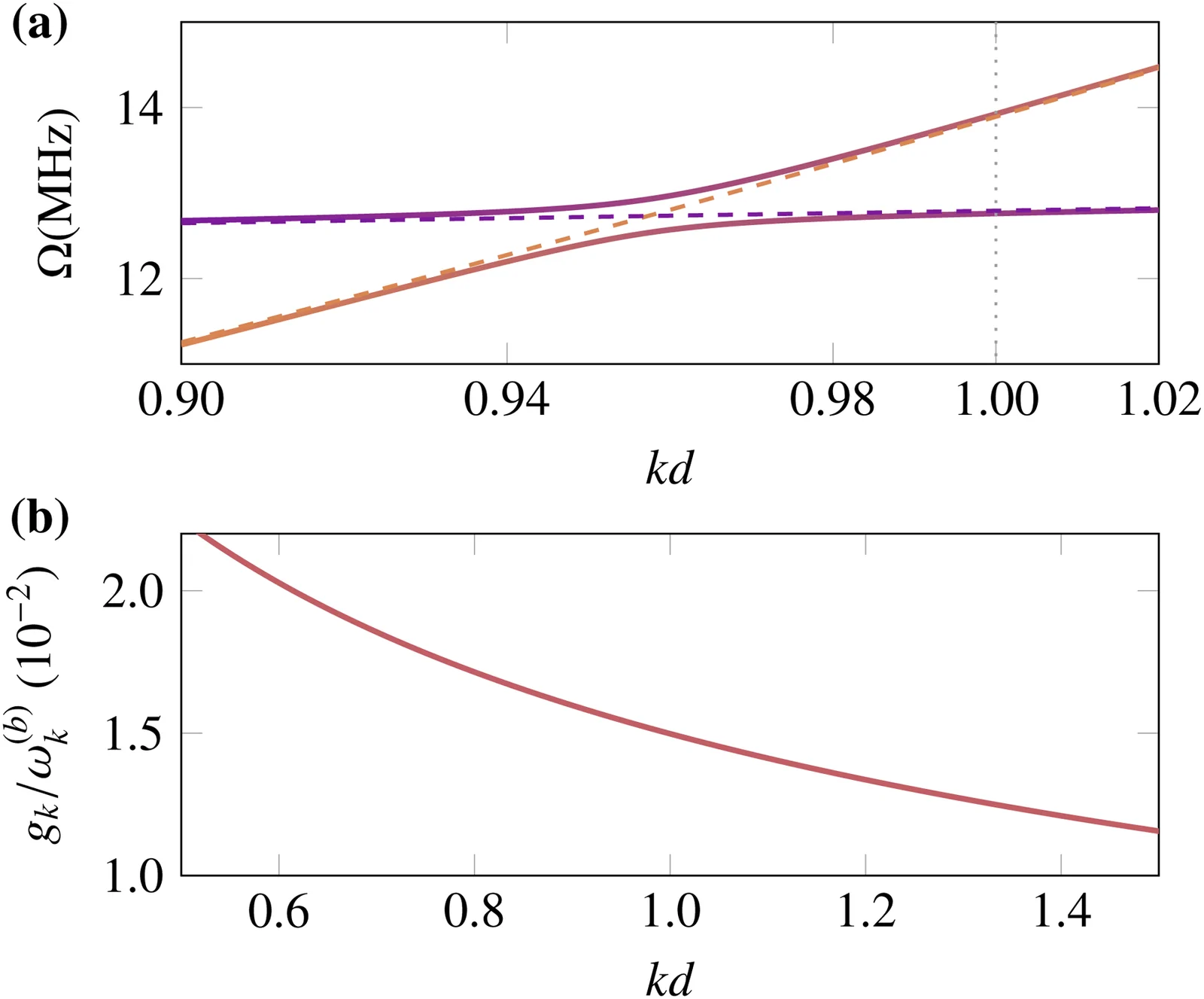
(Color online) (a) Dispersion relation from eqn (16) as a function of in-plane wave vector *kd* near the avoided-crossing region. Dashed lines indicate the bare flexural modes of graphene (lilac) and gold (orange), while solid lines correspond to the coupled upper and lower polariton branches. The vertical dotted line marks the representative point *kd* = 1, where *Ω*_UP_ = 2π × 13.92 MHz and *Ω*_LP_ = 2π × 12.76 MHz, corresponding to the upper and lower polariton modes, respectively. (b) Normalized coupling strength *g*_*k*_/*ω*^(*b*)^_*k*_ plotted *versus kd*. For these simulations, we considered the numerical parameters given in Table 1.

Fig. 2 illustrates the formation of hybridized polariton modes in the vicinity of the avoided-crossing region. As shown in Fig. 2(a), the bare dispersions (dashed lines) intersect near *kd* ≃ 0.96. When the Casimir-mediated coupling is included, this degeneracy is lifted, and an anticrossing appears, giving rise to well-defined upper and lower polariton branches (solid lines) with a splitting on the order of a few megahertz.

Fig. 2(b) displays the corresponding normalized coupling strength *g*_*k*_/*ω*^(*b*)^_*k*_ as a function of *kd*. The coupling decreases monotonically with increasing wave vector, indicating stronger hybridization in the long-wavelength regime. These results demonstrate coherent mode mixing and tunable coupling within the considered parameter range. The vertical dotted line indicates the representative wave vector *kd* = 1 used throughout the analysis, corresponding to flexural frequencies *ω*_*a*_ = 2π × 13.89 MHz and *ω*_*b*_ = 2π × 12.79 MHz, with a Casimir-induced coupling strength *g* = 2π × 0.2 MHz.

### B. Spectral response

We characterize the mechanical response of the system *via* the displacement spectrum17
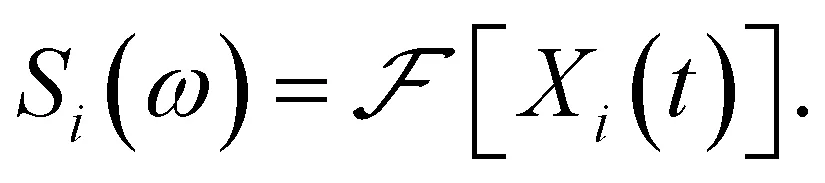
where *X*_*i*_(*t*) denotes the displacement of membrane *i* and 
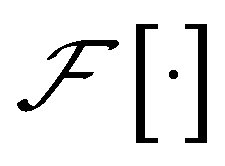
 is the Fourier transform.

**Fig. 3:**
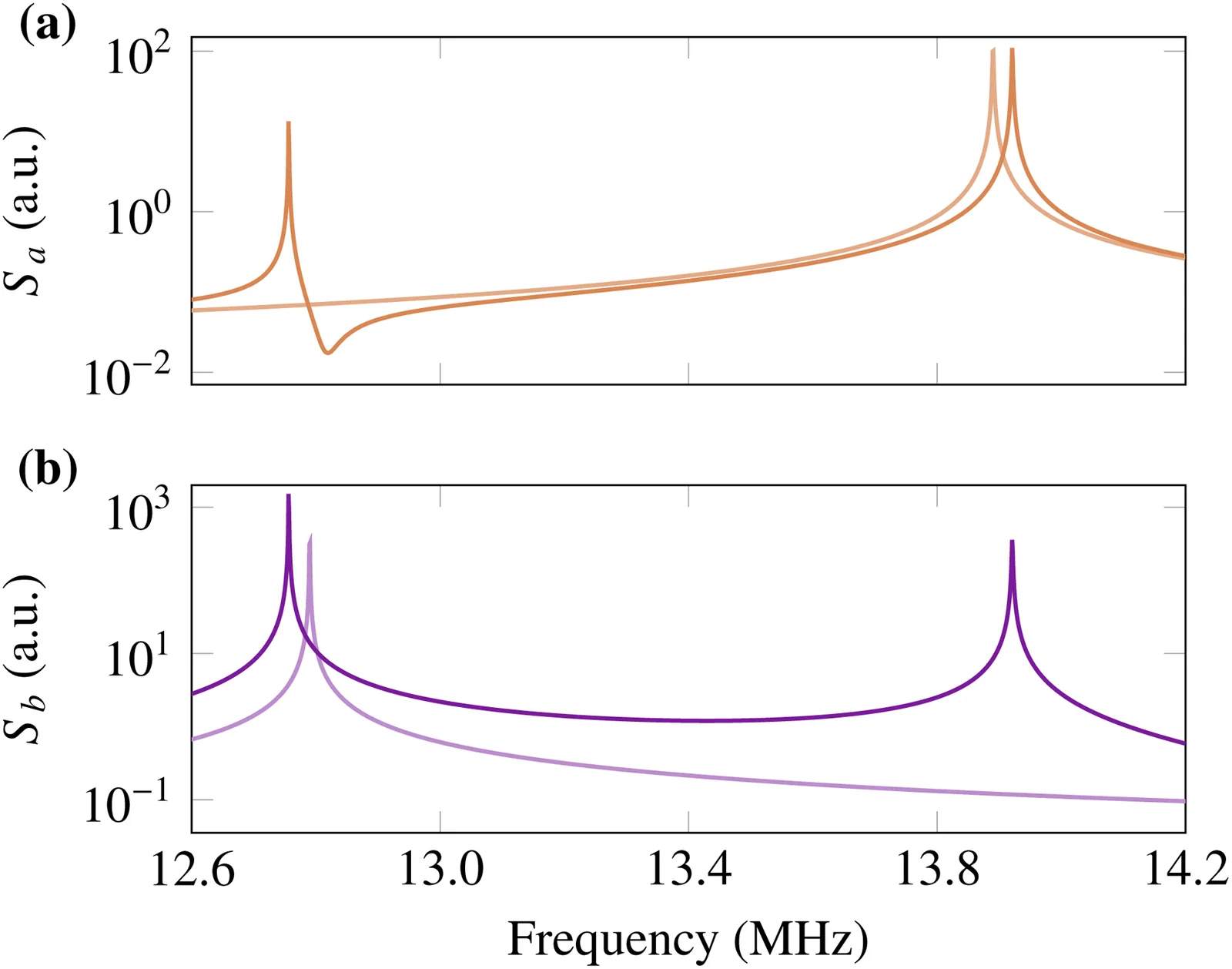
(Color online) Spectral comparison for (a) the gold resonator and (b) the graphene membrane. Spectra are shown for the uncoupled case (*g* = 0, lighter colors) and for the coupled case with *g* = 2π × 0.2 MHz (darker colors). In the uncoupled regime, the gold resonator spectrum *S*_*a*_ exhibits a peak at its bare frequency *ω*_*a*_ = 2π × 13.89 MHz, while the graphene membrane spectrum *S*_*b*_ peaks at *ω*_*b*_ = 2π × 12.79 MHz. When coupled, both spectra display two hybridized peaks: *ω*_*a*_ → *Ω*_UP_ = 2π × 13.92 MHz and *ω*_*b*_ → *Ω*_LP_ = 2π × 12.76 MHz, corresponding to the upper and lower polariton modes, respectively. For these simulations, we considered the numerical parameters given in Table 1.

In Fig. 3, we show the spectral response of the system for both uncoupled (free) and coupled membranes. The spectra reveal that the original flexural modes are shifted due to hybridization: *ω*_*a*_ → *Ω*_UP_ and *ω*_*b*_ → *Ω*_LP_ (see Fig. 2). Additionally, in the coupled regime the spectra exhibit cross-peaks corresponding to the flexural mode of the other membrane, a direct signature of Casimir-induced mode mixing.

**Fig. 4:**
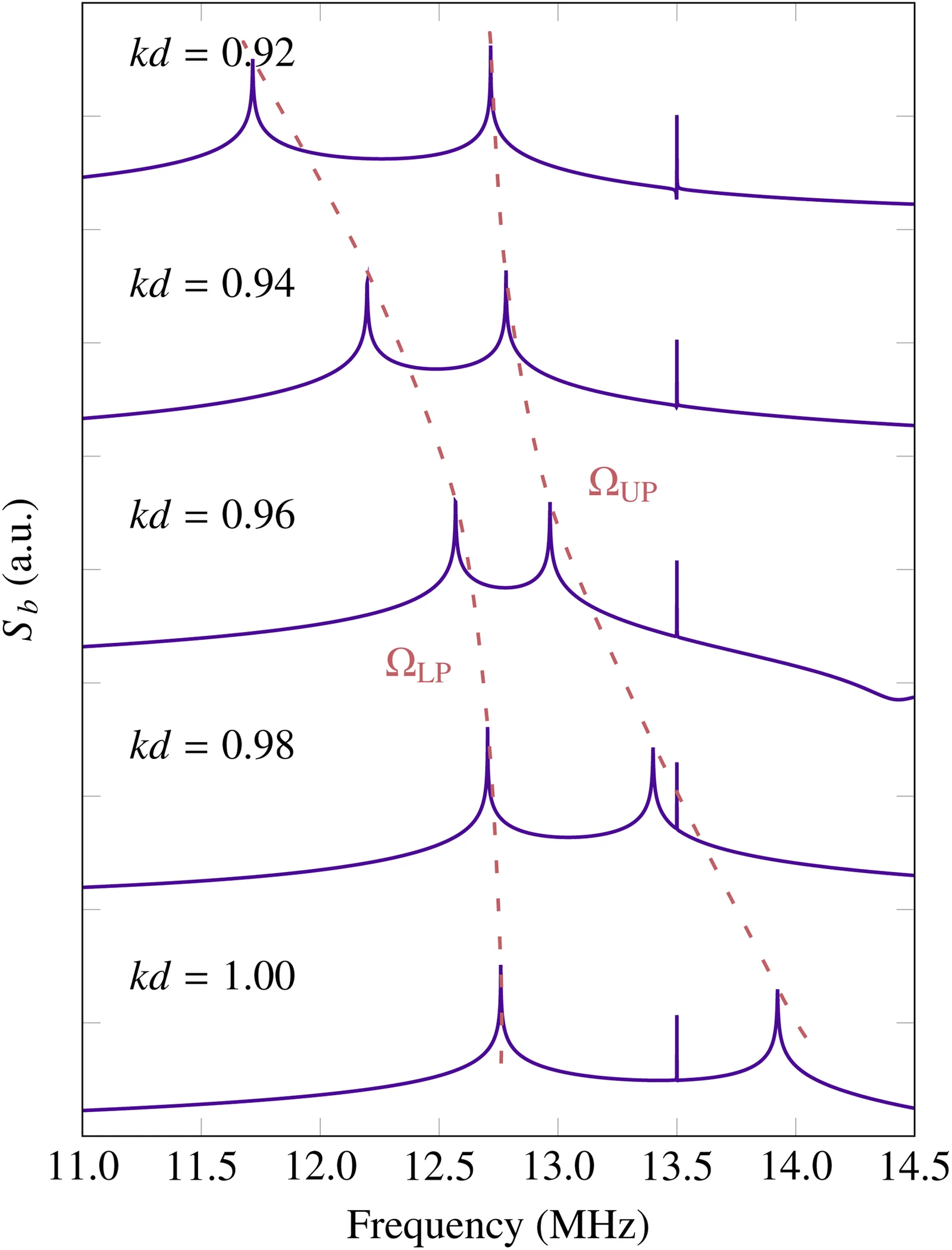
(Color online) Spectrum of the graphene membrane *S*_*b*_ under a driving force of 10 ms^−2^ at *ω*_d_ = 2π × 13.5 MHz. Variation of the spectral response with membrane separation reveals the Casimir-induced mode splitting. For these simulations, we considered the numerical parameters given in Table 1.

To experimentally probe the Casimir interaction, one can drive a single membrane (typically the gold-coated one) and measure the displacement spectra of both membranes at various separations. The drive is taken to be narrowband and tuned near a single hybridized polariton pair, and the mode spacing is large enough that off-resonant modes are weakly excited and can be neglected at leading order. Fig. 4 shows the spectral densities under such a configuration. Three peaks emerge: one at the drive frequency *ω*_d_, and two at the polariton frequencies *Ω*_UP_ and *Ω*_LP_. These can be used, together with eqn (16), to extract the coupling strength *g*_*k*_. As shown in Fig. 2, the polariton modes exhibit a gap as a function of in-plane wavevector *k*, while the normalized coupling strength *g*_*k*_/*ω*^(*b*)^_*k*_ quantifies the interaction regime. By tuning the membrane separation *d*, the system can access the strong- and ultrastrong-coupling regimes.

### C. Casimir-induced amplification

As a linear superposition of two different flexural modes, the lifetime of a polariton is directly determined by the decay rates of the graphene and gold modes, *Γ*_*b*_ ≡*Γ*_graph_ and *Γ*_*a*_ ≡*Γ*_gold_, as

18 *Γ* = |*u*_*k*_|^2^*Γ*_*b*_ + |*v*_*k*_|^2^*Γ*_*a*_,

where the Hamiltonian in eqn (6) has been diagonalized by introducing polariton operators

19 *A*_*k*_ = *u*_*k*_*a*_*k*_ + *v*_*k*_*b*_*k*_, *B*_*k*_ = −*u*_*k*_*a*_*k*_ + *v*_*k*_*b*_*k*_.

here *u*_*k*_ and *v*_*k*_ are the Hopfield coefficients, representing the gold- and graphene-like fractions of the hybrid mode (see ref. 15 for more details). The decay rates *Γ*_*i*_ can be estimated from the quality factors of the nanomechanical resonators *via Γ*_*i*_ = *ω*_*i*_/*Q*_*i*_.

In the following, Fermi's golden rule is used as a heuristic tool to quantify when Casimir-mediated energy transfer from the driven gold mode to the graphene mode exceeds its intrinsic loss, without implying fully quantum-coherent single-phonon processes. The underlying dynamics are classical: both modes are highly occupied, and the rates should be interpreted as stimulated classical scattering rates.

The scattering rate for a transition |*N*_*k*_〉 → |*N*_*k*_ + 1〉, corresponding to the emission of a flexural excitation in graphene, is20
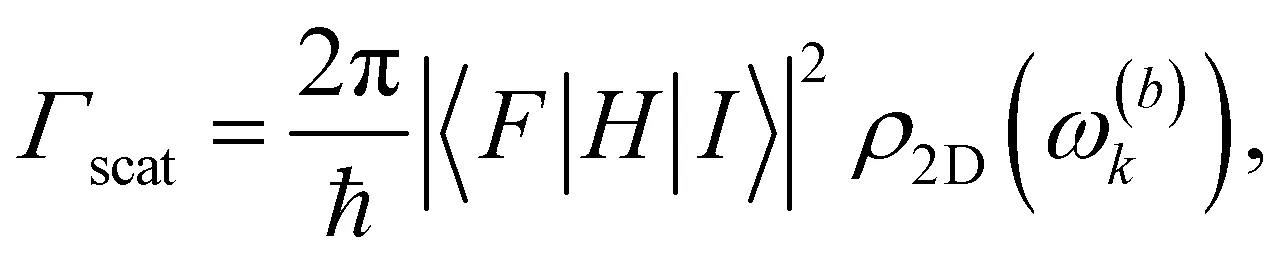
where *ρ*_2D_(*ω*) is the two-dimensional density of states^38^21
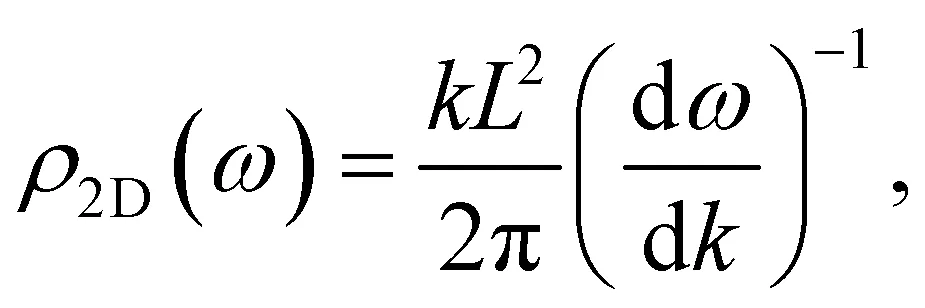
for a membrane of area *L*^2^. Retaining only the relevant terms in eqn (6) that mediate conversion from gold to graphene modes, one obtains

22 *Γ*_scat_ = 2π(*n*^(*b*)^_*k*_ + 1)*n*^(*a*)^_*k*_|*u*_*k*_|^2^|*v*_*k*_|^2^|*g*_*k*_|^2^*ρ*_2D_(*ω*^(*b*)^_*k*_),

where *n*^(*j*)^_*k*_ = [exp(*ℏω*^(*j*)^_*k*_/*k*_B_*T*) − 1]^−1^ is the Bose–Einstein distribution.

**Fig. 5:**
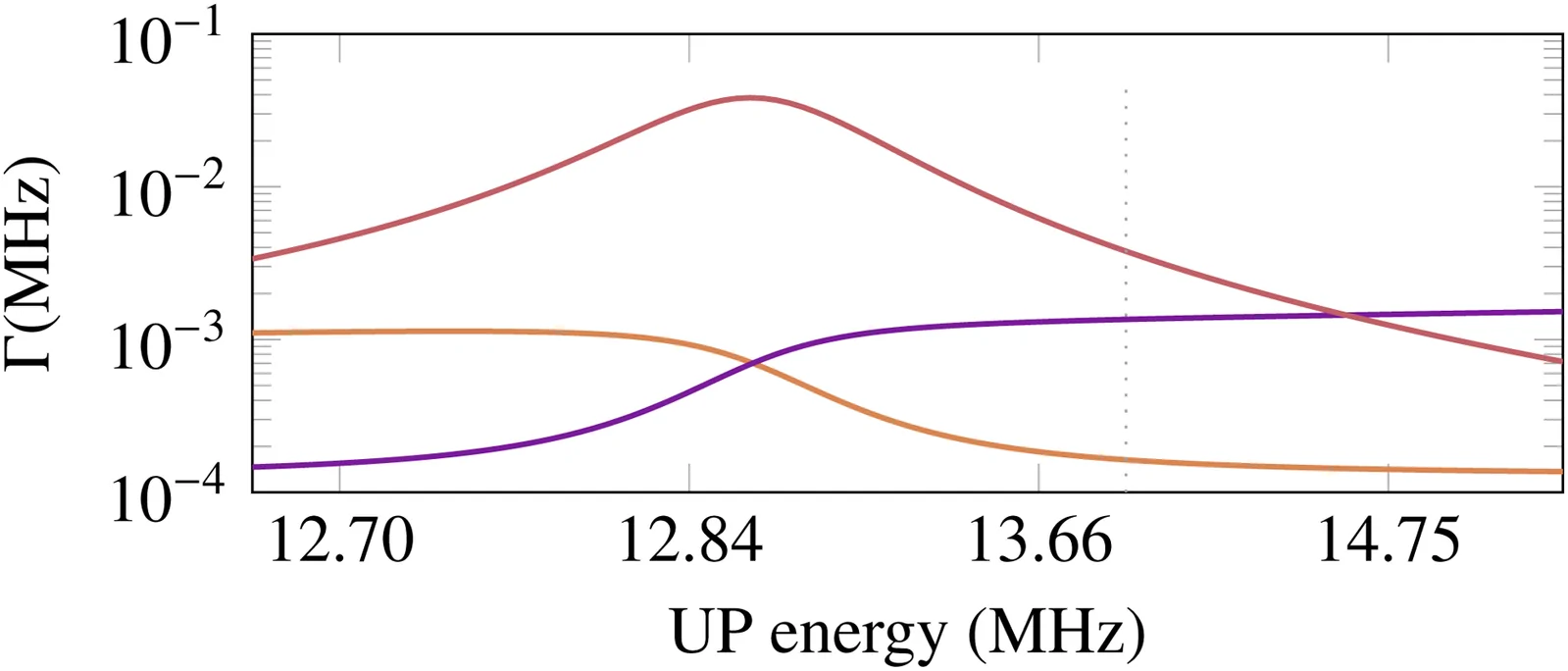
(Color online) Scattering decay rate into the final signal mode from the pump as a function of in-plane wave vector (pink), compared with the loss rates of the lower polariton (LP, orange) and upper polariton (UP, lilac) modes.

In the near-resonant regime depicted in Fig. 2, the scattering rate becomes comparable to the intrinsic loss rates of the modes. As shown in Fig. 5, the emission process can even exceed losses near the hybridization gap, entering the regime of strong, drive-induced energy transfer.

The emergence of classical gain in the graphene membrane can be understood directly from the properties of the hybridized polariton modes. Near the anticrossing, the upper and lower polariton eigenfrequencies, *Ω*_UP_ and *Ω*_LP_, are separated by the hybridization gap

23 Δ*Ω* = *Ω*_UP_ − *Ω*_LP_.

The corresponding eigenmodes are coherent superpositions of the gold and graphene flexural modes, with relative amplitudes determined by the Hopfield coefficients *u*_*k*_ and *v*_*k*_. Consequently, the effective decay rate of a polariton mode is given by eqn (18), reflecting the weighted contributions of the intrinsic damping of each membrane.

When the gold membrane is externally driven near *Ω*_UP_, energy is coherently transferred to the graphene component *via* the Casimir-mediated coupling. The stimulated scattering rate, which increases with the amplitude of the gold driving field,

24 *Γ*_scat_ ∝ |*u*_*k*_|^2^|*v*_*k*_|^2^|*g*_*k*_|^2^*ρ*_2D_(*ω*_*b*_)

quantifies this transfer. In the regime *Γ*_scat_ ≳*Γ*_*b*_, the graphene mode experiences a net reduction of its effective loss under continuous drive, leading to a strong increase in its steady-state amplitude. This balance between hybridization-induced energy transfer and intrinsic losses sets the threshold for the observed amplification and explains the increase in graphene displacement amplitude and linewidth narrowing under resonant driving of the gold membrane. We emphasized that this effect is entirely classical: the graphene motion is amplified by energy transfer from the driven gold membrane through hybridization, rather than by true stimulated emission or population inversion. Accordingly, the mechanism is most accurately described as Casimir-mediated parametric amplification.

To assess the experimental feasibility of Casimir-mediated parametric amplification with the system parameters considered in this work, we estimate the condition under which the stimulated scattering rate exceeds the intrinsic mechanical loss of the graphene mode. Using the quality factors in Table 1, we obtain intrinsic decay rates25
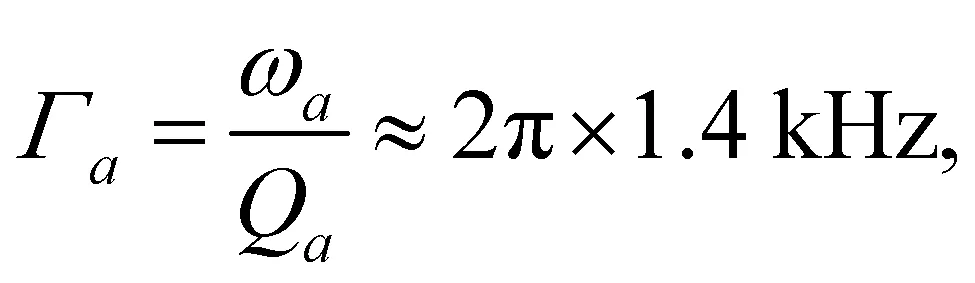
26
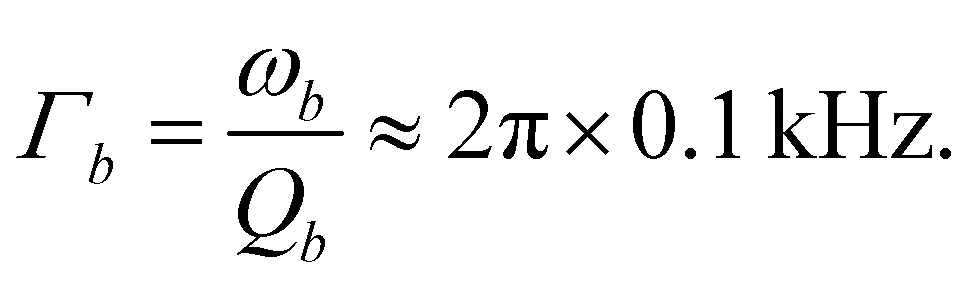


**Table 1:** Key parameters for experimental realization of the Casimir flexuron amplifier

Parameter	Symbol	Value
Membrane separation	*d*	1.5 µm
Gold film thickness	*δ*_Au_	20 nm
Gold Young's modulus	*E*_Au_	79 GPa
Gold Poisson ratio	*ν*_Au_	0.4
GaAs membrane thickness	*δ*_GaAs_	45 nm
GaAs Young's modulus	*E*_GaAs_	86 GPa
GaAs Poisson ratio	*ν*_GaAs_	0.31
Graphene bending rigidity	*D*_*b*_	1.5 eV
Graphene quality factor	*Q*_*b*_	1 × 10^5^
Au/GaAs quality factor	*Q*_*a*_	1 × 10^4^
Lateral membrane size	*L*	15 µm
Fundamental mode	*k*_0_	2π/15 µm
Operational wavevector	*k*	2π/(1.5 µm)
Flexural frequency gold	*ω*_*a*_	2π × 13.89 MHz
Flexural frequency graphene	*ω*_*b*_	2π × 12.79 MHz
Coupling strength	*g*	2π × 0.2 MHz

In the resonant driving regime near the upper polariton frequency, the stimulated scattering rate into the graphene mode can be estimated from eqn (22). The efficiency of this transfer is controlled by the hybridization coefficients *u*_*k*_ and *v*_*k*_, whose product |*u*_*k*_*v*_*k*_|^2^ is maximized near the avoided crossing, where |*u*_*k*_*v*_*k*_|^2^ ≃ 1/4, indicating strong mixing of the gold and graphene flexural modes.

At a temperature *T* = 100 mK, the thermal occupations of the mechanical modes follow from the Bose–Einstein distribution. Using *ℏω*_*b*_/*k*_B_*T* ≃ 0.0061 ≪ 1, we obtain*n*_*b*_ ≃ 164, *n*_*a*_≃150,so that both resonators are highly thermally populated and their dynamics can be treated in the classical regime.

The influence of the driven gold membrane on the graphene dynamics can be understood directly from the classical coupled-mode equations. Eliminating the driven-gold mode yields an effective equation for the graphene amplitude, in which the intrinsic damping rate *Γ*_*b*_ is modified by a coupling-induced correction proportional to *g*_*k*_^2^/*Γ*_*a*_. For near-resonant driving, the effective graphene damping rate takes the approximate form27
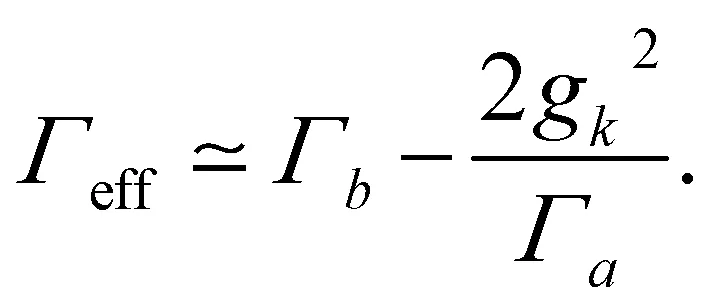


The apparent reduction of effective damping in the driven system corresponds to coherent energy transfer; in equilibrium, the Casimir-induced self-energy always increases damping. This expression shows that the Casimir-mediated coupling provides an additional channel for energy transfer from the driven gold resonator to the graphene mode.

A strong increase of the graphene displacement amplitude occurs when this transfer rate becomes comparable to or larger than the intrinsic graphene dissipation rate, *i.e.*,28
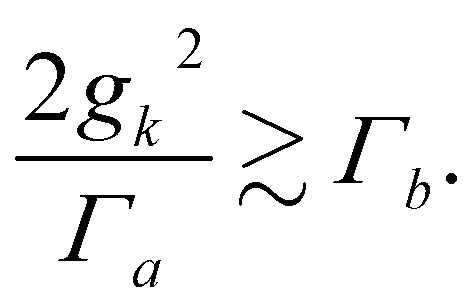


From eqn (25) and (26), we find that the Casimir-mediated transfer rate greatly exceeds the intrinsic damping of the graphene mode. Under moderate external driving, corresponding for example to a gold-mode population *n*_*a*_ ∼ 10^3^, the graphene motion can therefore be strongly enhanced through coherent energy transfer from the driven gold membrane. As *n*_*b*_ ≫ 1, the system remains firmly in the classical regime, and the enhanced graphene response arises from driven energy transfer between the hybridized flexural modes rather than from quantum-stimulated emission processes. The resulting increase in the steady-state graphene amplitude and the associated linewidth narrowing thus reflect the reduction of the effective mechanical damping produced by Casimir-mediated hybridization.

This conclusion is consistent with the more detailed linear-response analysis presented in Appendix A.1, where the graphene effective damping is shown to remain positive, and the enhanced response arises solely from coherent energy transfer *via* the driven gold membrane.

**Fig. 6:**
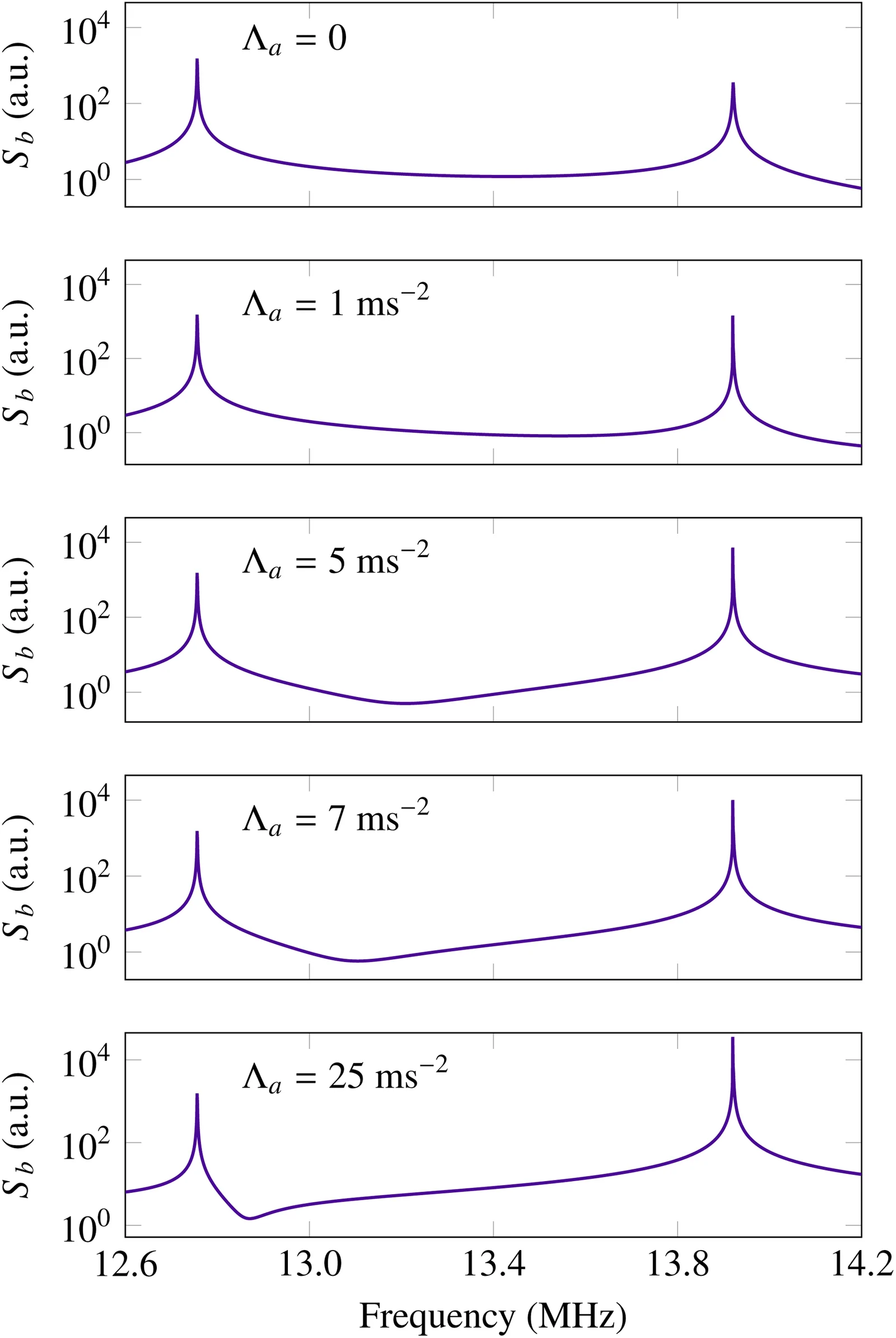
(Color online) Spectrum of the graphene membrane response to a driving force on the gold resonator, varied from 0 to 25 ms^−2^, at the upper polariton frequency *ω*_d_ = *Ω*_UP_. For these simulations, we considered the numerical parameters given in Table 1.

To illustrate this behavior, we numerically compute the output spectrum of the graphene membrane for increasing driving strengths applied to the gold membrane at *ω*_d_ = *Ω*_UP_. As shown in Fig. 6, the driven scattering process can exceed the intrinsic mechanical losses near the hybridization gap, leading to a strong enhancement of the graphene flexural response. The onset of a sharp increase in the graphene spectral amplitude is determined by the balance between external drive strength, intrinsic mechanical damping, and Casimir-mediated coupling. This behavior is characteristic of driven parametric amplification in coupled mechanical systems. Moreover, the linewidth of the emitted signal narrows below *Γ*_signal_/2π, consistent with coherent amplification of a selected mode. The observed spectral narrowing reflects phase-coherent amplification under resonant driving and is consistent with classical parametric gain mediated by hybridized mechanical modes.

## III. Energy transfer in Casimir-coupled nanomembranes

While our analysis demonstrates a pronounced enhancement of graphene flexural motion *via* Casimir-mediated hybridization with the driven gold membrane, the intrinsic damping of the graphene mode remains strictly positive under equilibrium conditions. It is therefore important to emphasize that the observed enhancement does not correspond to intrinsic gain or negative damping in the sense of a self-oscillating mechanical mode. Instead, the system operates in a driven, dynamically stable regime, where coherent energy transfer reshapes the response of the hybridized flexural modes without violating equilibrium stability conditions.

This distinction is well established in the theory of open nanomechanical systems: true negative damping and self-sustained oscillations require genuinely non-equilibrium conditions, such as thermal bias between reservoirs or explicit time-dependent modulation of system parameters.^39–41^ Related non-equilibrium concepts have been actively explored in nanoscale systems, including near-field energy transfer, fluctuation-induced forces, and non-contact dissipation, which are central themes in recent *Nanoscale* literature.^42,43^ A detailed discussion of these mechanisms and their relation to the graphene self-energy is provided in Appendix A.

Starting from the coupled equations of motion for the two membranes, elimination of the gold mode yields an effective graphene response of the form

29 [*ω*_*b*_^2^ − *ω*^2^ − i*Γ*_*b*_*ω* − *Σ*(*ω*)]*X*_*b*_(*ω*) = *F*_eff_(*ω*),

where *Σ*(*ω*) = *κ*_*a*_*κ*_*b*_*χ*_*a*_(*ω*) is the Casimir-induced self-energy, *F*_eff_(*ω*) is an effective drive, and the susceptibility of the gold membrane is given by30
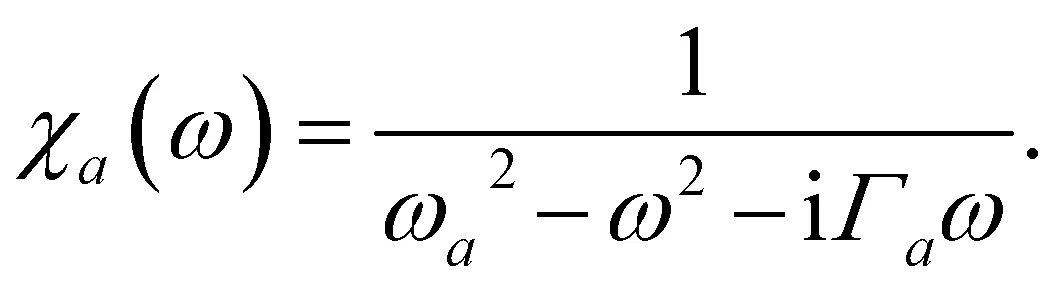


The imaginary part of the Casimir-induced self-energy reads31
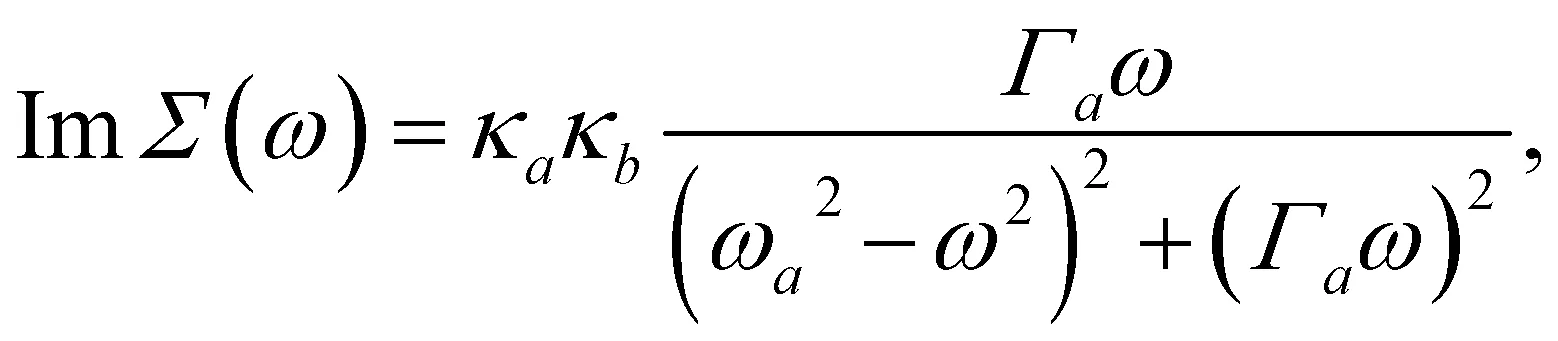
which is strictly positive. As a consequence, the effective damping of the graphene mode becomes^39,40^32
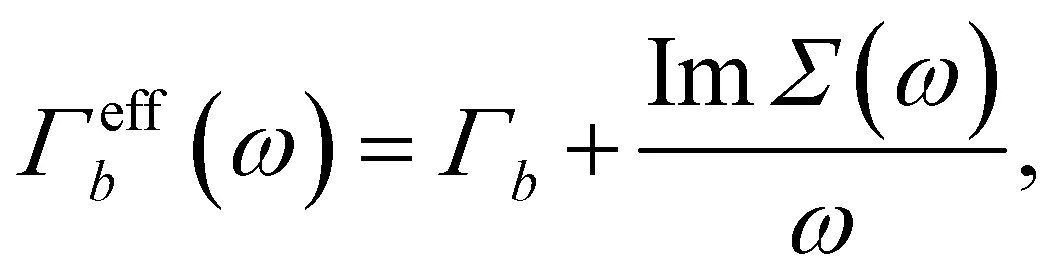
and remains positive in equilibrium. The equilibrium Casimir coupling therefore increases the total dissipation and reshapes the driven response through mode hybridization, but does not by itself generate self-sustained oscillations. The system is dynamically stable, and the observed amplification arises from coherent redistribution of energy injected by the external drive.

### A. Non-equilibrium regime

When the two membranes are coupled to reservoirs at different temperatures, *T*_*a*_ ≠ *T*_*b*_, detailed balance is broken and a net energy flux can develop through the Casimir-mediated coupling. In this situation, the graphene mode experiences a non-equilibrium pumping rate *Γ*_NEQ_ that partially compensates intrinsic damping. As derived in Appendix A.2, the non-equilibrium contribution at the graphene resonance takes the form33
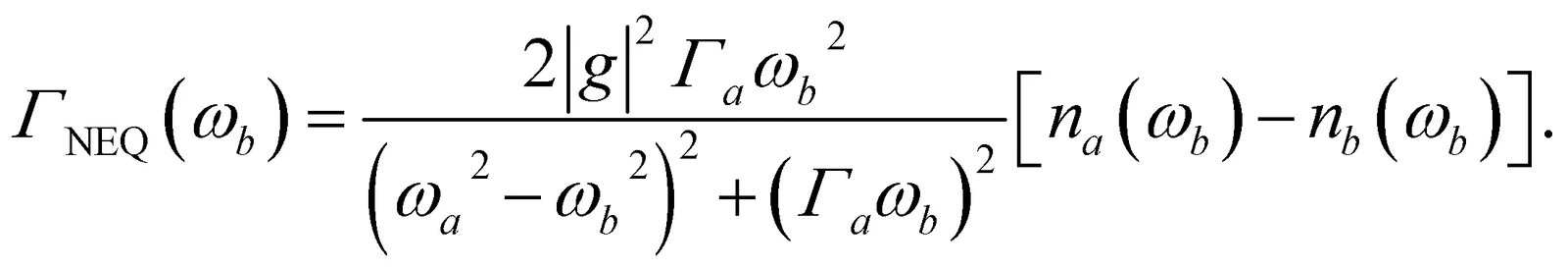


The total effective damping is then34
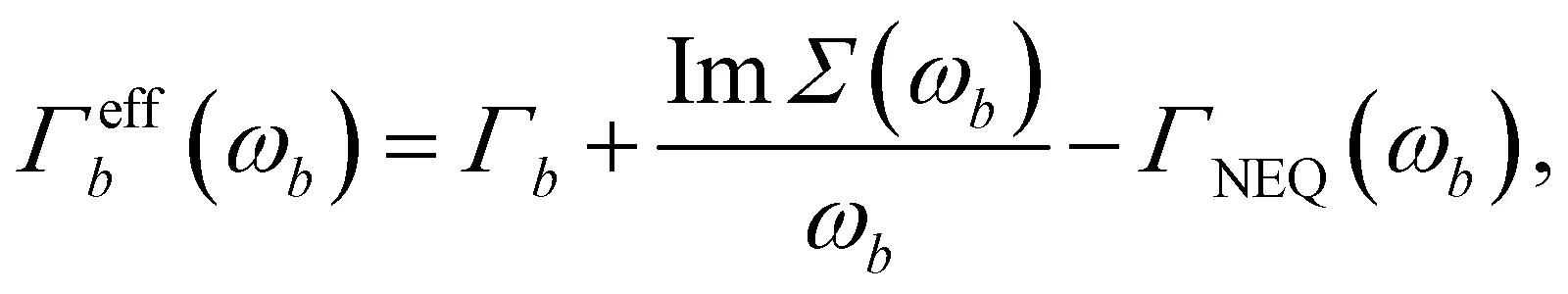
where the first two terms represent intrinsic and equilibrium Casimir-induced dissipation, while the last term corresponds to non-equilibrium pumping. For *T*_*a*_ > *T*_*b*_, one has *n*_*a*_(*ω*_*b*_) > *n*_*b*_(*ω*_*b*_), so that *Γ*_NEQ_ > 0 and the effective damping is reduced. If this pumping exceeds the sum of loss channels, *Γ*^eff^_*b*_ can become negative, triggering self-sustained oscillations.

The instability threshold is reached when35
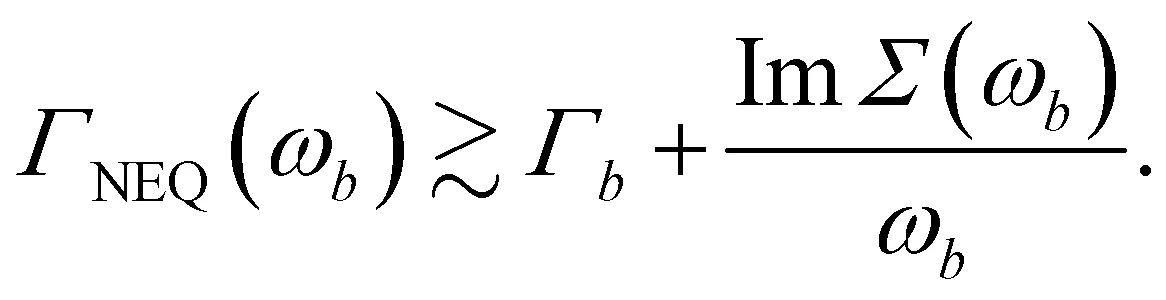


Near the hybridization point *ω*_*a*_ ≃ *ω*_*b*_, this condition simplifies to36
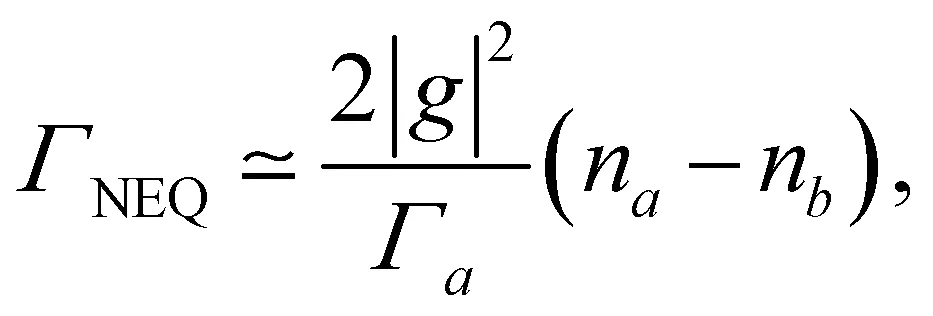
leading to the approximate threshold condition37
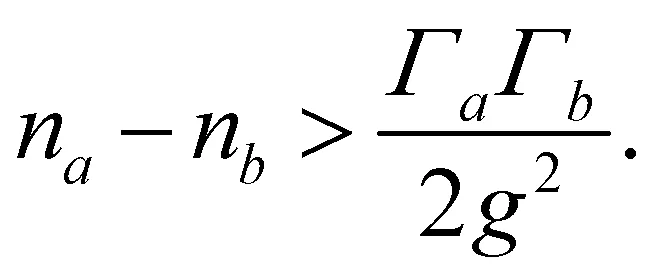


Using the experimental parameters considered here, the threshold occupation difference becomes

38 *n*_*a*_ − *n*_*b*_ ≳ 2 × 10^−6^.

At *T* = 100 mK, the mechanical occupations are deep in the classical regime, *n*_*j*_ ≃ *k*_*B*_*T*/(*ℏω*_*j*_) ≫ 1, corresponding to a formal temperature difference *T*_*a*_ − *T*_*b*_ ≳ 10^−9^ K.

This unrealistically small value reflects the simplicity of the single-mode, perfectly matched bath model. In realistic nanomechanical platforms, additional dissipation channels, imperfect spectral overlap, and finite mode selectivity substantially increase the non-equilibrium bias required to reach instability. Nevertheless, this estimate illustrates that Casimir-coupled membranes provide a sensitive pathway for non-equilibrium energy injection once appropriate conditions are engineered.

Genuine negative damping and self-sustained oscillations would therefore require explicit non-equilibrium engineering, such as temperature gradients, time-dependent modulation of the inter-membrane separation, relative motion (quantum friction), or coupling to externally driven electromagnetic environments. Such mechanisms, actively discussed in the nanoscale literature,^42,43^ differ fundamentally from the driven equilibrium hybridization studied here. Our results thus clarify the separation between Casimir-mediated parametric enhancement in a driven equilibrium setting and true non-equilibrium gain in nanomechanical systems.

## IV. Experimental feasibility and physical applications

From an experimental perspective, observing Casimir-mediated amplification of flexural phonons requires stable separation control, high mechanical quality factors, low-noise displacement readout, and careful suppression of unwanted electrostatic and substrate-mediated cross-talk. These requirements are challenging but compatible with state-of-the-art MEMS/NEMS, suspended-membrane, and cryogenic optomechanical platforms. The aim of the present work is therefore to establish the physical principle and the expected linear-response signatures of Casimir-mediated flexural-mode coupling, while also identifying realistic experimental constraints.

We focus primarily on low-temperature operation, where thermal mechanical noise is reduced, and high mechanical quality factors can be maintained. Unless stated otherwise, the numerical estimates below correspond to temperatures *T* ≲ 100 mK and micrometer-scale separations. In this regime, the amplification dynamics are governed primarily by the Casimir-induced coupling between the flexural modes, while thermal phonons enter mainly through the intrinsic mechanical damping rates and the displacement noise floor. A full treatment of temperature-dependent Casimir corrections and high-temperature phonon transport is beyond the scope of this work. We expect elevated temperatures to renormalize the effective coupling, damping, and noise floor, without qualitatively altering the Casimir-mediated mode hybridization discussed here.

### A. Path to experimental realization

A possible implementation is illustrated schematically in Fig. 1. The device may be fabricated from two independently prepared suspended membranes assembled in a face-to-face geometry. A high-*Q* GaAs micromechanical or nanomechanical membrane can be fabricated using standard semiconductor-processing and sacrificial-etch techniques and then coated with a thin Au layer. The Au coating enhances the electromagnetic response entering the Casimir interaction, while the GaAs platform provides a mature mechanical support compatible with external actuation, optical readout, and piezoelectric driving. Separately, a graphene monolayer can be transferred onto a substrate with a predefined trench or circular aperture, forming a suspended graphene resonator. The two chips can then be aligned and positioned using lithographic spacers, wafer bonding, or piezoelectric nanopositioning.

The choice of graphene and Au-coated GaAs is motivated by their complementary properties. Graphene has an extremely low areal mass density, strong out-of-plane flexural response, high sensitivity to mass and force loading, and an established role in nanomechanical sensing. The Au layer increases the Casimir coupling through its strong electromagnetic response, while GaAs offers a robust and well-developed nanomechanical platform. The mechanism is not restricted to this specific material pair: other high-*Q* suspended two-dimensional materials, metallic membranes, polaritonic membranes, or semiconductor resonators could also be considered. The graphene/Au-coated GaAs platform should therefore be viewed as a representative and physically motivated implementation rather than as the only possible design.

In the proposed scheme, the Au-coated GaAs membrane is driven piezoelectrically at a frequency *ω*_d_ close to one of the hybridized normal-mode resonances. Through the Casimir-mediated displacement–displacement coupling, energy injected into the Au/GaAs membrane is coherently transferred to the graphene flexural mode. The motion of the two membranes can be detected using independent interferometric readout channels, focused near the membrane centers to maximize sensitivity to the fundamental flexural modes. The relevant experimental signatures are avoided crossings in the hybridized spectrum, linewidth modification, and drive-dependent enhancement of the graphene displacement response.

Observability requires that the Casimir-mediated signal exceed the thermal displacement noise. The thermal displacement-noise spectral density of the graphene membrane is^44,45^39
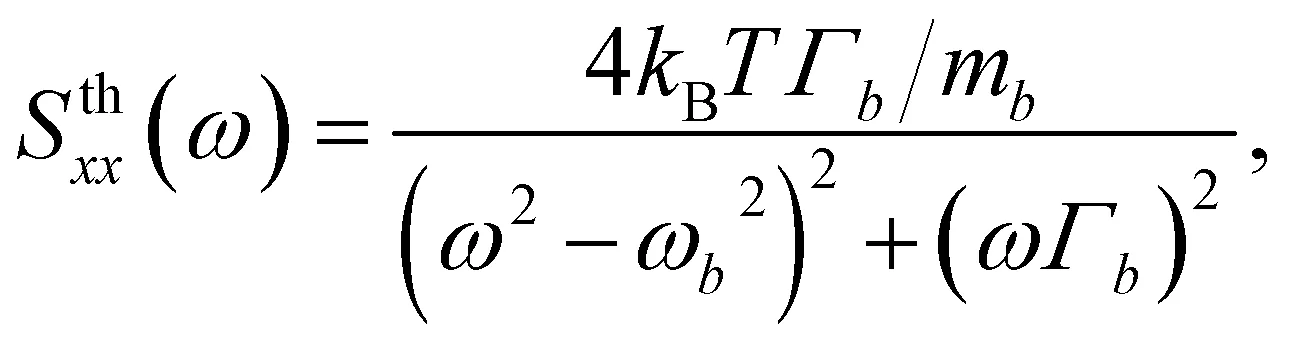
with *T* the base temperature, *m*_*b*_ is the effective mass of the graphene mode, and *Γ*_*b*_ is its intrinsic damping rate. For parameters in Table 1 and *T* = 100 mK, the thermal noise floor near *ω*_d_ is *S*^th^_*xx*_ ∼ 10^−30^ m^2^ Hz^−1^, whereas the coherent response gives a displacement variance 〈*X*_*b*_^2^〉 ∼ 10^−18^ m^2^ (see Fig. 6). For a 1 Hz measurement bandwidth, this corresponds to SNR ≫ 1.

The drive force required to reach the onset of drive-induced amplification, estimated from *Γ*_scat_ ≳*Γ*_*b*_, is *F*_th_ ∼ 10^−15^ N. This value is well within the range of standard piezoelectric actuation. In typical nanomechanical experiments, piezoelectric actuators driven with millivolt-to-volt excitation voltages produce base displacements in the range *x*_p_ ∼ 10^−11^ to 10^−9^ m, providing a convenient and experimentally accessible method for driving micro- and nanomechanical resonators. For a membrane with effective mass *m*_*a*_, the resulting inertial drive force *F*_d_ ≃ *m*_*a*_*ω*_d_^2^*x*_p_ can reach 10^−14^ to 10^−12^ N for MHz-frequency resonators, exceeding the threshold force by several orders of magnitude.^46–48^ This suggests that the required drive strength is experimentally accessible. Increasing the membrane area further improves the signal-to-noise ratio because the coherent Casimir force grows with the mode-overlap area, whereas uncorrelated noise contributions add incoherently.

### B. Gap dependence, alignment tolerances, and supported graphene

The membrane separation *d* is the central experimental control parameter. For ideal parallel metallic plates, the Casimir pressure scales as *P*_Casimir_ ∝ *d*^−4^. In the present mechanical problem, the relevant quantity is the induced dynamical coupling rate *g*(*d*) between the two flexural modes. In the perturbative regime considered here, this coupling inherits a strong algebraic dependence on the separation, approximately*g*(*d*) ∝ *d*^−4^.

Consequently, the hybrid-mode splitting scales as *g*(*d*), while the resonant energy-transfer rate in the weak-coupling regime has the generic Golden-rule scaling^15^*Γ*_transfer_ ∝ *g*(*d*)^2^ ∝ *d*^−8^.

Thus, even modest changes in separation can strongly affect the mode hybridization and energy-transfer efficiency.

A nominal separation of *d* ≃ 1.5 µm provides a compromise between coupling strength and experimental stability. At smaller separations, especially *d* ≲ 500 nm, electrostatic patch potentials, residual charges, surface roughness, pull-in, and stiction become increasingly important and can introduce unwanted nonlinearities or instability.^49,50^ At larger separations, for example, *d* ≳ 5 µm, the Casimir-mediated coupling is strongly reduced and may no longer overcome intrinsic mechanical losses. The micrometer regime therefore provides a practical balance between measurable coupling and mechanical stability.^51^

The relevant requirement is stable control of the average separation over the mode-overlap area and over the mechanical measurement time, rather than atomic-scale control of the absolute separation. Optical interferometry, capacitance measurements, piezo-controlled approach curves, or the observed hybrid-mode splitting can be used to calibrate the effective gap and coupling strength *in situ*. Lateral misalignment and non-parallelism reduce the spatial mode-overlap factor and therefore reduce the effective coupling, but they do not change the qualitative mechanism provided the two flexural modes retain appreciable spatial overlap.

Electrostatic interactions arising from residual charges, patch potentials, or applied voltages primarily induce static position shifts or modest renormalization of mechanical resonance frequencies through electrostatically induced tension.^33,42^ Such effects are well known in suspended nanomembrane and graphene-based nanoelectromechanical systems, where electrostatic forces are routinely used for tuning and actuation rather than as sources of dynamical gain 35. In the present proposal, the amplification mechanism does not require electrostatic modulation. Residual electrostatic backgrounds can be reduced by grounding, charge compensation, and independent characterization of the distance dependence. Moreover, electrostatic cross-talk can be distinguished experimentally from Casimir-mediated hybridization by its different dependence on separation, bias voltage, and detuning.

Several practical aspects are important for experimental implementation:

(i) Vacuum and temperature. Ultra-high vacuum suppresses gas damping and supports high quality factors,^52^ while cryogenic operation reduces thermal noise and helps access the low-temperature non-equilibrium regime considered here.^53,54^

(ii) Membrane geometry. Lateral dimensions of *L* ∼ 10–20 µm provide sizable mode-overlap areas while preserving experimentally accessible flexural frequencies and high mechanical quality factors.

(iii) Separation control and mechanical isolation. Nanometer-precision piezoelectric nanopositioners, as routinely used in Casimir-force and near-field optomechanics experiments, can set and stabilize the target separation.^55^ Fabricating the membranes on independent chips helps suppress direct mechanical cross-talk, so that the dominant interaction occurs across the vacuum gap;^56,57^ residual vibrations can be quantified by off-resonant driving or detuning the separation.

(iv) Alignment and surface effects. Small deviations from parallelism, surface roughness, and finite membrane curvature modify the spatially averaged coupling strength^58,59^ but they do not qualitatively alter the existence of Casimir-mediated mode hybridization. The Casimir interaction remains tunable *via* the average separation to compensate for imperfections.

(v) Reproducibility and observables. Avoided crossings in hybridized spectra, linewidth narrowing of the graphene mode under drive, and drive-dependent enhancement of displacement spectral density provide clear and reproducible signatures of Casimir-mediated energy transfer without requiring absolute force calibration.

(vi) Suppression of direct cross-talk. Direct vibration transfer from the piezo-driven Au/GaAs membrane to graphene can be minimized by mounting graphene on an independent substrate or a secondary suspended platform. Phononic band-gap structures and multi-stage suspension further suppress substrate-mediated vibrations.^60^ Residual cross-talk is distinguishable by its weaker distance dependence and off-resonant response compared with the strongly resonant Casimir-mediated interaction.

If graphene is supported directly on a substrate, the free flexural spectrum is substantially modified. The substrate can gap or damp the out-of-plane modes, reduce the mechanical quality factor, introduce additional loss channels, and alter the electromagnetic boundary conditions entering the Casimir interaction. Fully suspended graphene is therefore the most favorable configuration for observing strong Casimir-mediated flexural-mode hybridization. Nevertheless, partially suspended or cavity-supported graphene geometries may offer a practical compromise for sensing applications, provided that a well-defined high-*Q* out-of-plane mechanical mode remains.

Operating in the micrometer-separation regime with high-*Q* membranes, precise separation control, and careful mechanical isolation thus provides a realistic route to observing Casimir-mediated hybridization and drive-induced amplification of flexural phonons, enabling controlled, non-contact energy transfer between nanoscale mechanical resonators.

### C. Representative sensing application

As a representative application, we consider mass sensing with the graphene flexural mode, following the standard principle of resonant NEMS mass sensing.^61–63^ The resonance frequency of a mechanical mode can be written as40
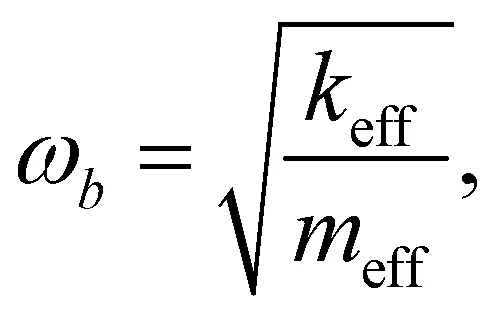
where *k*_eff_ and *m*_eff_ are the effective spring constant and effective modal mass of the graphene flexural mode. Adsorption of a small mass *δm*, such as a gas molecule, biomolecule, or nanoparticle, changes the effective mass of the graphene resonator and shifts its resonance frequency. If the adsorption does not significantly modify the membrane tension or bending rigidity, then *k*_eff_ remains approximately unchanged, while *m*_eff_ → *m*_eff_ + *δm*. Therefore,41
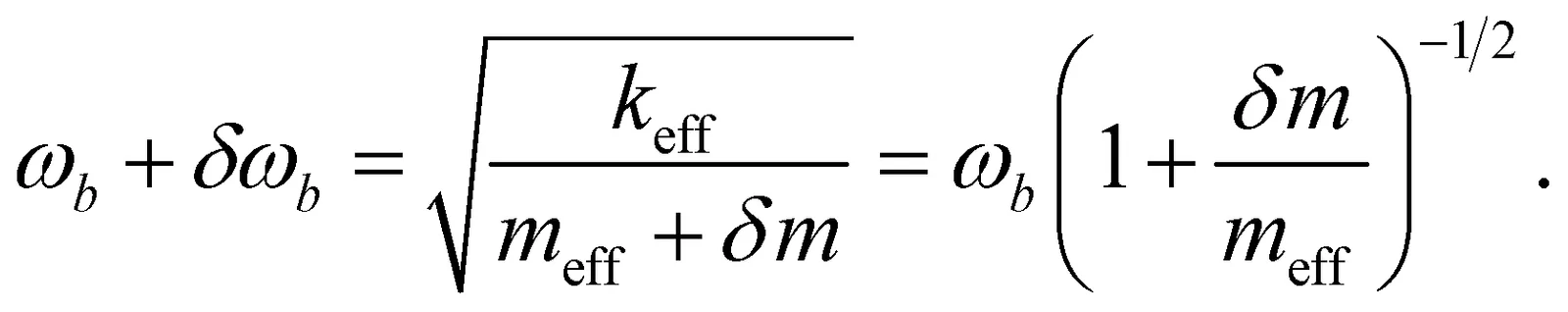


For *δm* ≪ *m*_eff_, the leading frequency shift is42
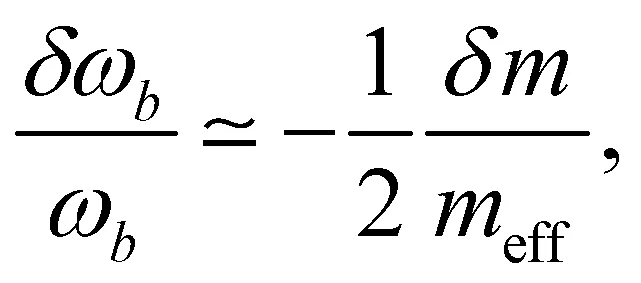


The minimum detectable mass is obtained by replacing the frequency shift in eqn (42) by the smallest resolvable frequency shift, *δω*_min_. Thus,43
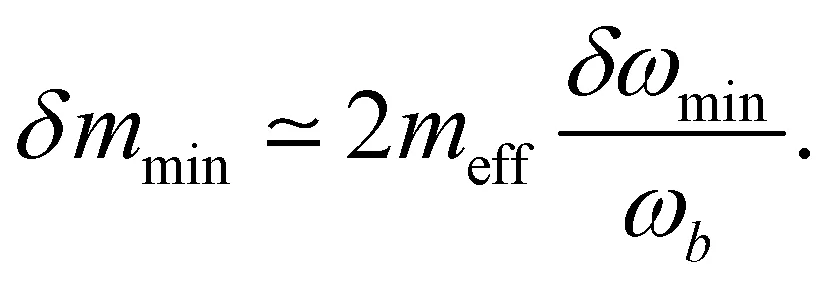


Near the hybridized resonance, Casimir-mediated coupling can enhance the driven displacement response of the graphene mode and modify its effective linewidth. For a fixed displacement readout noise floor, a larger coherent response improves the signal-to-noise ratio and can reduce the smallest resolvable frequency shift *δω*_min_. In this way, Casimir-mediated flexural-mode hybridization can improve mass or force sensing by converting weak perturbations of the graphene mechanical frequency into enhanced displacement signals. If the measured resonance is a hybridized mode rather than the bare graphene mode, the observed frequency shift is weighted by the graphene fraction of that hybrid mode.

A quantitative limit of detection requires device-specific information, including adsorption geometry, displacement readout noise, thermal force noise, nonlinear damping, frequency noise, and environmental fluctuations. Such a full noise analysis is beyond the scope of the present work. The scaling in eqn (42) and (43), however, shows how the proposed Casimir-enhanced graphene response can be incorporated into realistic mass-sensing protocols.

### D. Comparison with existing coupling and amplification schemes

It is useful to compare the proposed Casimir-mediated coupling with more established resonator-coupling and amplification mechanisms. Electrostatic coupling can be strong and voltage-tunable, and it is widely used for actuation and tuning in graphene and other nanomechanical resonators.^64,65^ However, it requires nearby electrodes or bias voltages and can introduce charge noise, patch-potential forces, dielectric loss, and additional dissipation. Piezoelectric coupling is highly efficient for actuation and signal transduction, especially in materials such as GaAs, AlN, or LiNbO_3_,^66,67^ but it requires integration with piezoelectric media, and is not directly available for pristine graphene. Optomechanical and microwave electromechanical platforms provide sensitive readout, coherent control, and amplification of mechanical motion,^68,69^ but they require optical or microwave cavities and may introduce heating or additional technical noise.

Casimir-mediated coupling is complementary to these approaches. It is non-contact and voltage-free, and it can couple two mechanically separated membranes across a vacuum gap without requiring a direct electrical, elastic, or optical link. Casimir forces have been used and proposed for actuation, nonlinear dynamics, and coupling in micro- and nanomechanical systems.^15,70^ The main limitations of the present mechanism are its comparatively weak magnitude, strong dependence on separation, sensitivity to surface and electrostatic backgrounds, and the need for high-*Q* suspended membranes with stable gap control. The advantage of the Casimir mechanism is therefore not that it universally exceeds electrostatic, piezoelectric, or optomechanical coupling strengths, but that it provides a distinct vacuum-mediated channel for coherent mechanical energy transfer and amplification.

Although the static Casimir interaction is not switchable in the same way as a voltage-controlled electrostatic force, the effective energy transfer can be controlled dynamically by tuning the drive amplitude and frequency of the Au-coated GaAs membrane, by adjusting the membrane separation, and by tuning the detuning between the two flexural resonances. This provides functional control of the hybridized response and enables mode-selective amplification of graphene flexural motion.

### E. Broader physical applications

Beyond the representative mass-sensing example above, amplification of out-of-plane vibrational modes in graphene and other atomically thin membranes is relevant to several areas:

(i) Mass and force sensing. Flexural modes are highly sensitive to local mass changes (*e.g.*, molecular adsorption) and weak forces. Amplifying the graphene response can improve signal-to-noise ratios in graphene-based NEMS sensors for chemical and biological detection.^71,72^

(ii) Mechanical signal processing. Controlled excitation of out-of-plane modes enables dynamic tuning of the resonant frequency, crucial for frequency-selective devices and RF filters.^73,74^

(iii) Thermal transport. Flexural phonons carry a significant fraction of thermal energy in graphene. Controlling their amplitude and population allows detailed studies of phonon–phonon scattering and engineering of thermal conductivity for nanoscale thermal management.^38,75^

(iv) Quantum and hybrid optomechanics. Out-of-plane graphene modes can couple to photons, electrons, and other mechanical resonators.^76–79^ Controlled Casimir-mediated amplification could provide a route to studying vacuum-mediated energy transfer, phonon gain, and decoherence in suspended mechanical systems.

(v) Structural mechanics of two-dimensional materials. Flexural modes govern thermal ripples, wrinkling, and out-of-plane deformations in freestanding graphene. Controlling these modes may therefore provide a tool for studying mechanical stability and nonlinear membrane dynamics.

(vi) Optomechanical platforms. Out-of-plane vibrations modulate cavity resonance and light intensity; amplified flexural modes enhance light–matter interaction, facilitating modulators, photonic sensors, and light-controlled oscillators.

Although the Casimir force is static and not directly switchable like a voltage-controlled electrostatic interaction, it offers features difficult to reproduce electrostatically. It is long-range and quantum-coherent, enabling non-contact coupling without electrode-induced dissipation or decoherence; it naturally mediates mode-selective amplification of specific flexural modes; and it provides a platform for exploring fluctuation-induced energy transfer and, in future low-noise implementations, quantum thermomechanical effects. Moreover, the effective intermode coupling can be tuned *via* the amplitude and frequency of the externally driven gold membrane, providing functional “switchability” of the amplification. Casimir-based devices are therefore complementary to conventional actuation and ideal for exploring quantum-coherent phonon dynamics.

Finally, the present framework can be extended to multiple membranes with strong coupling between neighbors (*e.g.*, *via* substrate-mediated interactions), in analogy with the platform proposed in ref. 80. That work demonstrates a fully integrated, tunable mechanical array of high-Q resonators that can be parametrically driven and strongly coupled, enabling experimental studies of complex collective dynamics and analog computing paradigms such as Ising-type optimization, neuromorphic processing, and many-body nonlinear physics. Similar functionality could be realized in our system by engineering coupling *via* vacuum fluctuations.

### F. Relation to near-field radiative and phonon-assisted heat transfer

It is useful to place the present mechanism in the broader context of near-field energy transfer across vacuum gaps. Previous work has considered radiative heat transfer between graphene and other two-dimensional Dirac materials within fluctuational electrodynamics.^81^ In that case, energy is transferred by electromagnetic near-field fluctuations, including evanescent and plasmonic modes, and the relevant material properties are the optical conductivities or reflection coefficients of the two surfaces. The mechanical flexural motion of the membranes is not treated as an independent resonant degree of freedom.

A related but distinct mechanism was analyzed by Pendry *et al.*,^30^ who studied phonon-assisted heat transfer between vacuum-separated surfaces mediated by van der Waals forces. In their model, a phonon incident on one surface produces a periodic surface displacement, which generates a time-dependent van der Waals force on the opposing surface. This force can transmit phononic energy across the vacuum gap. They showed that this contribution is strongly distance-dependent and compared it with conventional near-field radiative heat transfer.

The mechanism studied here is complementary to both of these approaches. Rather than computing the total broadband heat flux between two passive media, we focus on the coherent coupling of two selected flexural modes: one in the Au/GaAs membrane and one in the suspended graphene membrane. The Casimir interaction produces an effective displacement–displacement coupling,

44 *H*_int_ = *g*_*k*_(*a*_*k*_ + *a*^†^_*k*_)(*b*_*k*_ + *b*^†^_*k*_),

which hybridizes the two mechanical modes and produces avoided crossings, linewidth modification, and resonant energy transfer. Under non-equilibrium conditions, the same coupling can reduce the effective damping of the graphene mode and, above threshold, lead to amplification.

Thus, the near-field energy exchange in such systems may contain several physically distinct channels,

45 *J*_total_ ≃ *J*_EM_ + *J*^vdW/Casimir^_ph_ + *J*^flex^_coh_,

where *J*_EM_ denotes the electromagnetic radiative channel studied in ref. 81, *J*^vdW/Casimir^_ph_ denotes the broadband phonon-assisted contribution considered in ref. 30, and *J*^flex^_coh_ denotes the resonant, mode-selective flexural channel analyzed in the present work. The last contribution is not simply a broadband thermal heat flux; it is a coherent mechanical energy-transfer process that is enhanced when the two flexural resonances are tuned close to one another. A quantitative comparison of these channels for a specific device geometry is beyond the scope of the present work, but the comparison highlights that Casimir-mediated flexural-mode coupling provides an additional channel not captured by a purely electromagnetic near-field heat-transfer calculation.

## V. Conclusions

We emphasize that the amplification described here arises in a driven, open-system setting. The energy required for gain is supplied externally *via* the piezoelectric drive applied to the Au/GaAs membrane, while the Casimir interaction provides a mode-selective, non-radiative coupling mechanism between the two membranes mediated by vacuum electromagnetic fluctuations. Without Casimir-mediated coupling, driving the gold membrane alone does not lead to amplification of graphene flexural modes; conversely, the Casimir interaction by itself does not supply energy. The observed energy transfer and enhancement of graphene's flexural motion result from the interplay between coherent driving and Casimir-induced mode hybridization, which occurs when the coherent Casimir-mediated coupling rate exceeds the intrinsic mechanical dissipation rates, placing the system in the strong-coupling regime.

We have developed a harmonic-oscillator framework that compactly captures this Casimir-enabled coherent energy transfer and resonant enhancement of flexural phonons in a suspended graphene membrane coupled to a driven gold-coated GaAs membrane. The Hamiltonian formulation is fully equivalent to the classical coupled equations of motion and allows exact diagonalization of the bilinear interaction, providing direct insight into the mode hybridization that underlies the observed parametric amplification. Our results demonstrate the feasibility of harnessing Casimir forces as a static coupling resource for coherent energy transfer and mode-selective amplification in nanoscale mechanical systems, opening the way for phononic devices that leverage vacuum-field interactions in a driven, classical regime.

While graphene offers an ideal platform due to its low mass density, strong flexural response, and compatibility with Casimir coupling, the mechanism described here is generic to suspended two-dimensional membranes supporting flexural modes. The essential ingredients are a soft out-of-plane mode, a high mechanical quality factor, and proximity to a driven mechanical resonator. Thus, the flexuron amplification concept applies broadly to other 2D materials and nanomechanical membranes.

In summary, we have shown that out-of-plane phonon modes in graphene can be coherently driven and resonantly amplified *via* mode hybridization through Casimir-mediated coupling, providing direct access to the flexural dynamics intrinsic to its two-dimensional nature. This capability enables new approaches for engineering graphene's mechanical and thermal properties, as flexural modes dominate its low-frequency phonon spectrum and significantly influence both thermal conductivity and membrane stability. Amplifying these flexural modes enhances graphene's potential as a platform for ultra-sensitive mass and force sensing, mechanically tunable resonators, and phonon-mediated optomechanical coupling.

Furthermore, controlled excitation of out-of-plane modes offers a route to investigate nonlinear phonon interactions, classical-quantum crossover effects, and the role of flexural phonons in out-of-equilibrium transport. Combined with the non-equilibrium scenarios discussed, our findings establish flexural mode control as a key mechanism for functionalizing graphene and related 2D materials in next-generation phononic, thermal, and quantum nanodevices, and delineate the boundary between equilibrium Casimir-mediated parametric enhancement and genuinely non-equilibrium negative damping.

## Conflicts of interest

There are no conflicts to declare.

## Appendix A. Negative damping in Casimir-coupled membranes

### 1. Equilibrium Casimir coupling and effective damping

We consider the linearized dynamics of two coupled membranes, a gold-coated membrane and a graphene membrane, in the presence of intrinsic damping. An external drive is applied only to the gold membrane. The equations of motion are

A1 *Ẍ*_*a*_ + *Γ*_*a*_*Ẋ*_*a*_ + *ω*_*a*_^2^*X*_*a*_ = *κ*_*a*_*X*_*b*_ + *Λ*_*a*_ cos(*ω*_d_*t*),

A2 *Ẍ*_*b*_ + *Γ*_*b*_*Ẋ*_*b*_ + *ω*_*b*_^2^*X*_*b*_ = *κ*_*b*_*X*_*a*_,

where *X*_*a*_ and *X*_*b*_ are the displacements of the gold and graphene membranes, *ω*_*a*,*b*_ are their natural frequencies, *Γ*_*a*,*b*_ are intrinsic damping rates, *κ*_*a*,*b*_ are coupling constants, and *Λ*_*a*_ is the amplitude of the external drive.

Fourier transforming to frequency space and defining the gold membrane susceptibilityA3
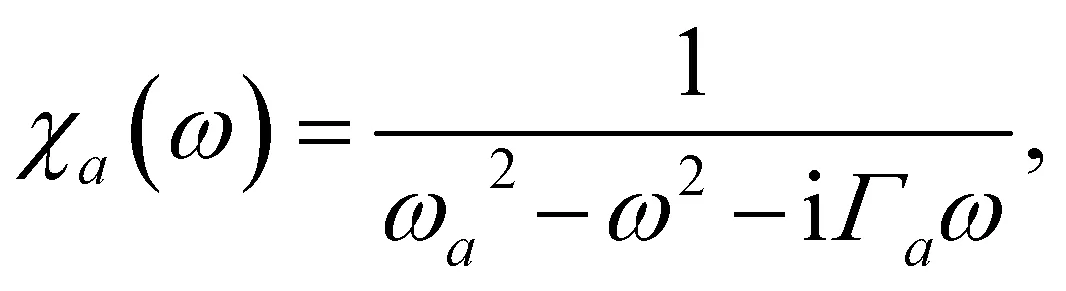
we eliminate *X*_*a*_(*ω*) to obtain an effective equation for the graphene mode

A4 [*ω*_*b*_^2^ − *ω*^2^ − i*Γ*_*b*_*ω*− *Σ*(*ω*)]*X*_*b*_(*ω*) = *κ*_*b*_*χ*_*a*_(*ω*)*Λ*_*a*_*δ*(*ω* − *ω*_d_),

with the Casimir-induced self-energy

A5 *Σ*(*ω*) = *κ*_*a*_*κ*_*b*_*χ*_*a*_(*ω*)

The conservative self-spring corrections discussed in Section I have been absorbed into the renormalized frequencies *ω*_*a*_ and *ω*_*b*_. They modify the real part of the inverse susceptibility but do not add an imaginary contribution proportional to −i*ω* in the static equilibrium regime. Therefore, they shift the resonance condition but do not change the sign of the damping. The damping modification discussed here arises from the imaginary part of the retarded self-energy associated with coupling to the other mechanical mode and, in the non-equilibrium case, from the additional pumping term *Γ*_NEQ_.

The imaginary part of the self-energy *Σ*(*ω*) is strictly positiveA6
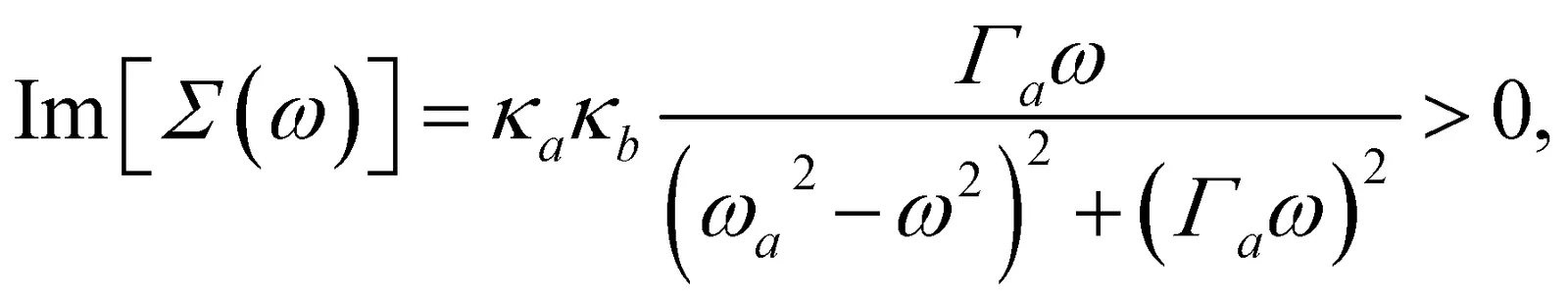
so that the graphene effective damping of the graphene mode,A7
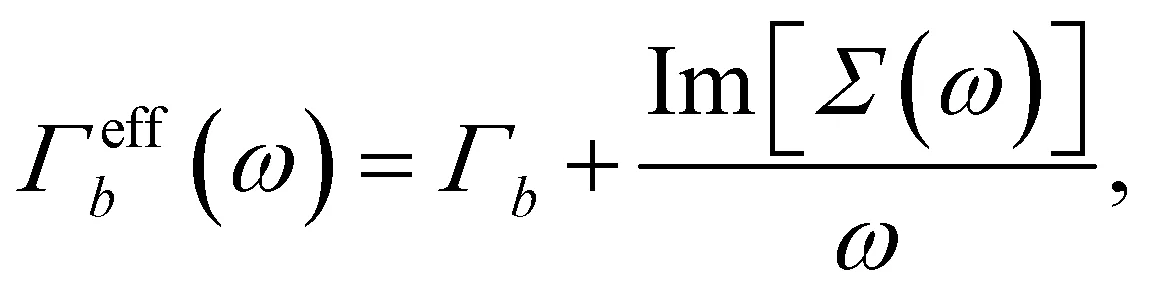
remains positive. This identification follows directly from standard linear-response and open-system treatments of harmonic oscillators coupled to baths.^39,40^

Consequently, an equilibrium Casimir coupling can enhance the driven response of the graphene membrane but does not produce self-sustained oscillations or intrinsic gain. Because Im[*Σ*(*ω*)] > 0, the coupling always adds damping rather than reducing it. Even when the gold membrane is externally driven on resonance, the graphene response is enhanced solely by coherent energy transfer *via* hybridization in the driven system, not by a sign change of the intrinsic damping.

### 2. General self-energy and effective damping

More generally, the frequency-domain equation for the graphene flexural mode can be written as

A8 [−*ω*^2^ − i*Γ*_*b*_*ω* + *ω*_*b*_^2^ − *Σ*(*ω*)]*X*_*b*_(*ω*) = *F*_drive_(*ω*),

where *Γ*_*b*_ is the intrinsic damping of the graphene mode, and *Σ*(*ω*) is the self-energy arising from the Casimir-mediated coupling to the gold membrane, and *F*_drive_(*ω*) is a generic external drive.

Introducing the Green functionA9
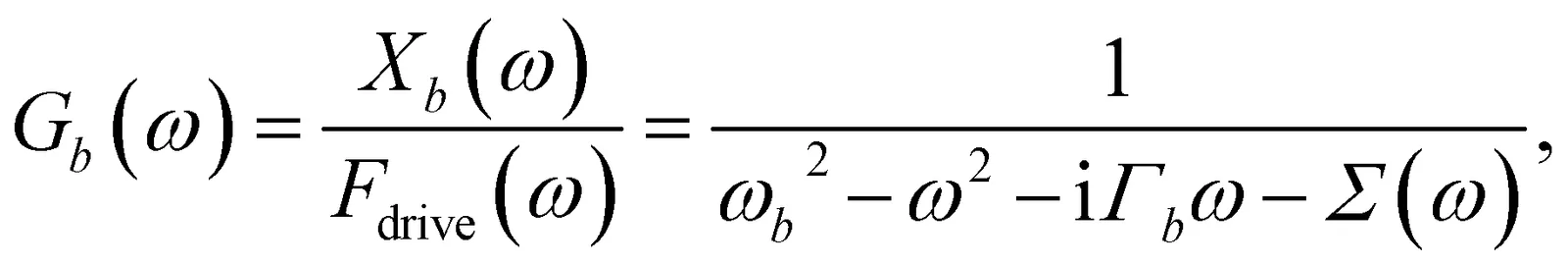
we identify the effective damping from the imaginary part of the denominator asA10
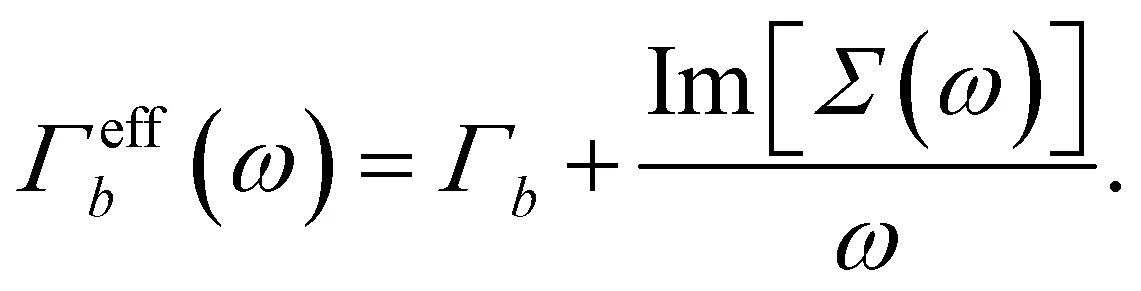


In non-equilibrium situations, for instance, when the two membranes are coupled to baths at different temperatures, *T*_*a*_ ≠ *T*_*b*_, or to a non-equilibrium electromagnetic environment, the self-energy acquires additional contributions,

A11 *Σ*_NEQ_(*ω*) = *Σ*_eq_(*ω*) + *Σ*^NEQ^_fluct_(*ω*),

where *Σ*^NEQ^_fluct_(*ω*) encodes the non-equilibrium fluctuations. In this case, the imaginary part can become negative,A12
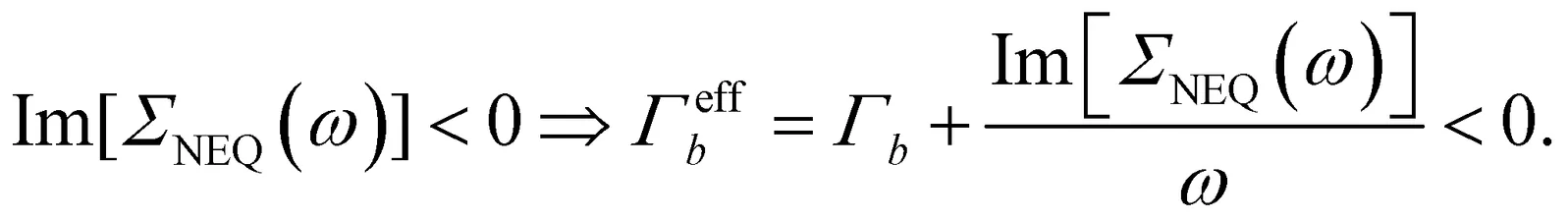


Physically, Im[*Σ*_NEQ_] < 0 corresponds to a mode-selective negative damping channel: energy is pumped preferentially into the graphene flexural mode. Once the non-equilibrium contribution exceeds the intrinsic damping,

A13 |Im[*Σ*_NEQ_(*ω*)]/*ω*| > *Γ*_*b*_,

self-sustained oscillations of the graphene mode become possible. This mechanism contrasts with equilibrium hybridization, where *Γ*^eff^_*b*_(*ω*) remains positive, and enhanced motion arises only from resonant energy transfer.

### 3. Non-equilibrium Casimir-induced negative damping and instability

We now analyze explicitly the conditions for self-oscillation of the graphene flexural mode driven by a temperature bias between the two membranes. We assume that the gold and graphene membranes are coupled to independent thermal reservoirs at temperatures *T*_*a*_ and *T*_*b*_, respectively, with *T*_*a*_ ≠ *T*_*b*_.

Restricting to a single in-plane wavevector *k*, the coupled Langevin equations of motion, including thermal fluctuations, are

A14 *Ẍ*_*a*_ + *Γ*_*a*_*Ẋ*_*a*_ + *ω*_*a*_^2^*X*_*a*_ = *κ*_*a*_*X*_*b*_ + *ξ*_*a*_(*t*),

A15 *Ẍ*_*b*_ + *Γ*_*b*_*Ẋ*_*b*_ + *ω*_*b*_^2^*X*_*b*_ = *κ*_*b*_*X*_*a*_ + *ξ*_*b*_(*t*),

where *ξ*_*a*_ and *ξ*_*b*_ are stochastic forces arising from the two independent baths. In equilibrium, the fluctuation-dissipation theorem gives
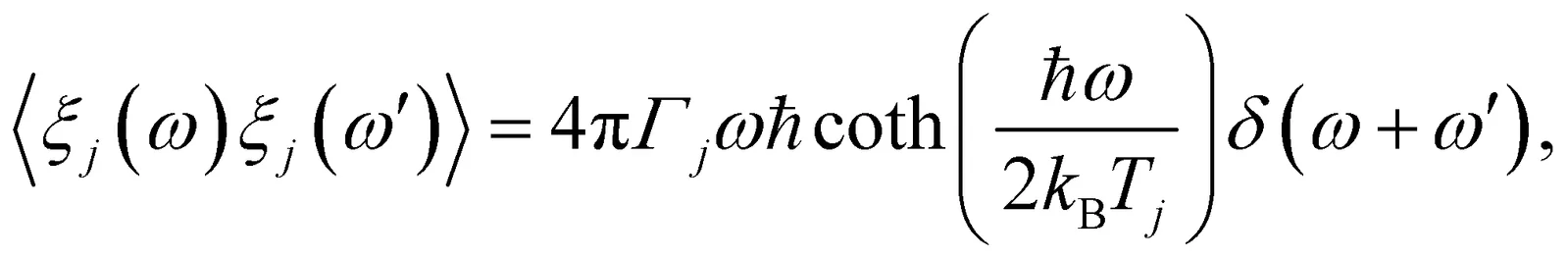
with *j* = *a*, *b*, 〈*ξ*_*a*_*ξ*_*b*_〉 = 0. In particular, one has 〈*ξ*_*j*_*ξ*_*j*_〉 ∝ *T*_*j*_, so that for *T*_*a*_ ≠ *T*_*b*_ the balance between fluctuations is broken.

Fourier transforming and introducing the gold susceptibilityA16
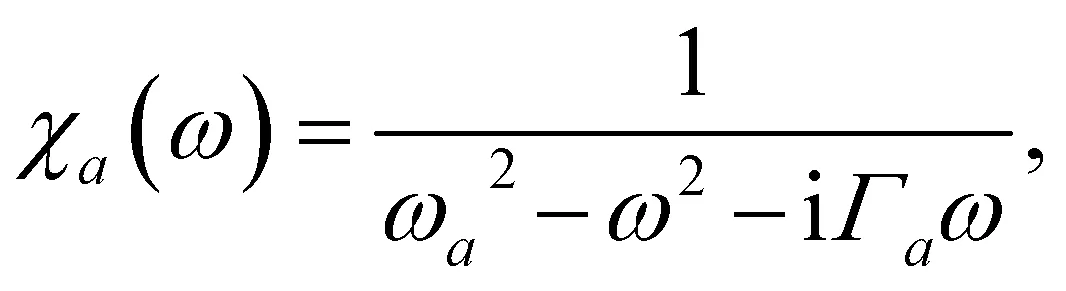
we eliminate *X*_*a*_(*ω*) as

A17 *X*_*a*_(*ω*) = *χ*_*a*_(*ω*)[*κ*_*a*_*X*_*b*_(*ω*) + *ξ*_*a*_(*ω*)].

Substitution into the graphene equation yields

A18 [*ω*_*b*_^2^ − *ω*^2^ − i*Γ*_*b*_*ω* − *Σ*(*ω*)]*X*_*b*_(*ω*) = *Ξ*_eff_(*ω*),

with self-energy

A19 *Σ*(*ω*) = *κ*_*a*_*κ*_*b*_*χ*_*a*_(*ω*),

and the effective stochastic force acting on graphene is

A20 *Ξ*_eff_(*ω*) = *κ*_*b*_*χ*_*a*_(*ω*)*ξ*_*a*_(*ω*) + *ξ*_*b*_(*ω*).

The corresponding graphene Green function isA21
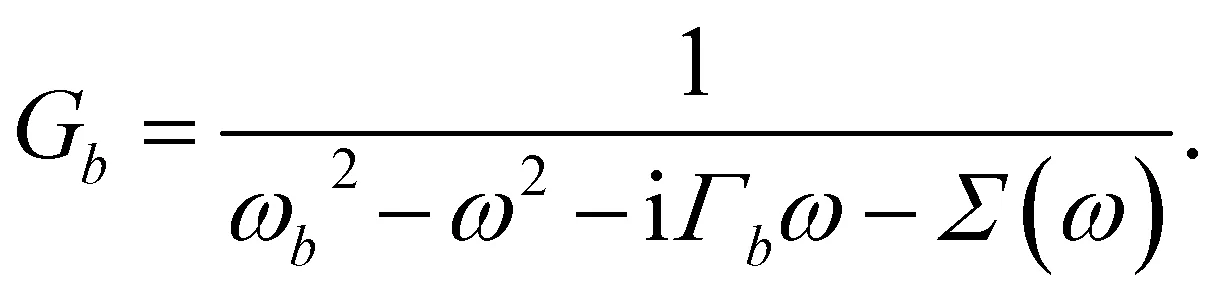


The imaginary part of the self-energy *Σ*(*ω*) isA22
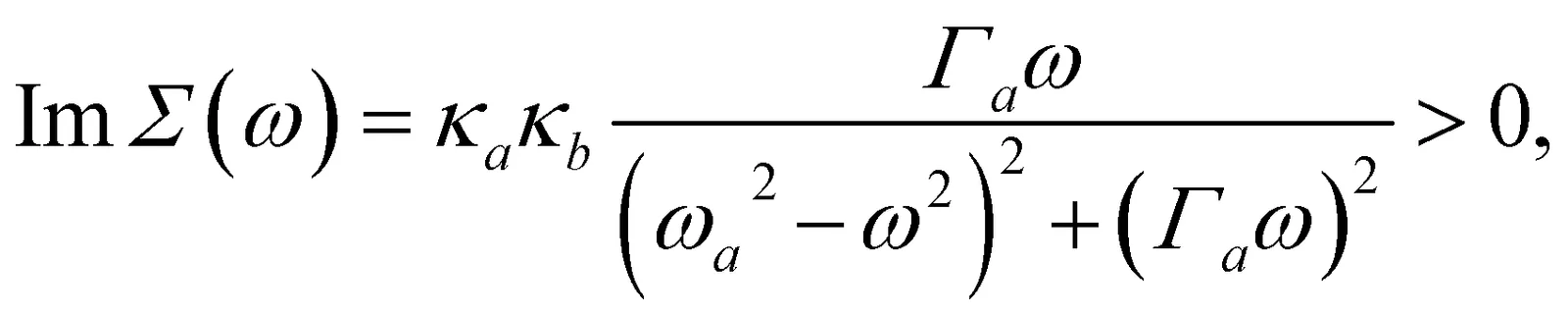
so that, at the level of the deterministic equations of motion, the equilibrium Casimir coupling always increases the damping of the graphene mode.

To quantify the non-equilibrium energy flow, we compute the rate at which work is done on the graphene mode by the Casimir coupling. The instantaneous power delivered to the graphene oscillator is

A23 *P*(*t*) = *Ẋ*_*b*_(*t*)*F*_int_(*t*),

where the interaction force exerted by the gold membrane is *F*_int_(*t*) = *κ*_*b*_*X*_*a*_(*t*). In the stationary state, the average power isA24
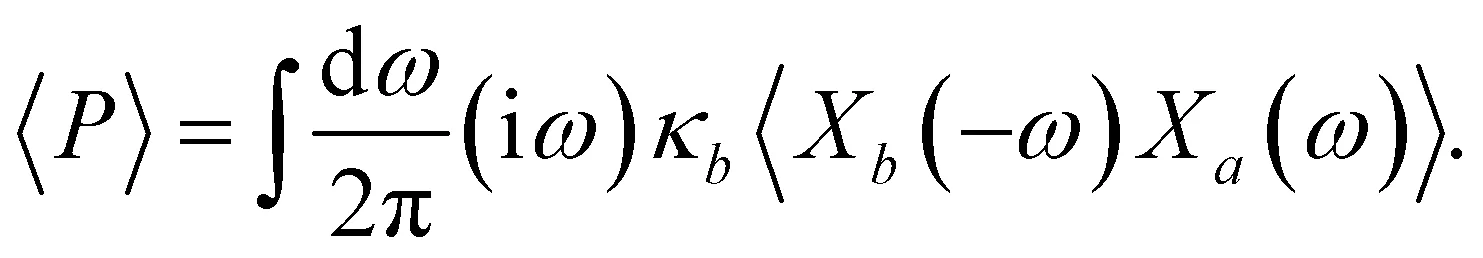


Using linear response, the displacements are expressed in terms of the stochastic forces *via* their susceptibilities. Substituting *X*_*a*_(*ω*) = *χ*_*a*_(*ω*)[*κ*_*a*_*X*_*b*_(*ω*) + *ξ*_*a*_(*ω*)], into eqn (A24), we obtainA25
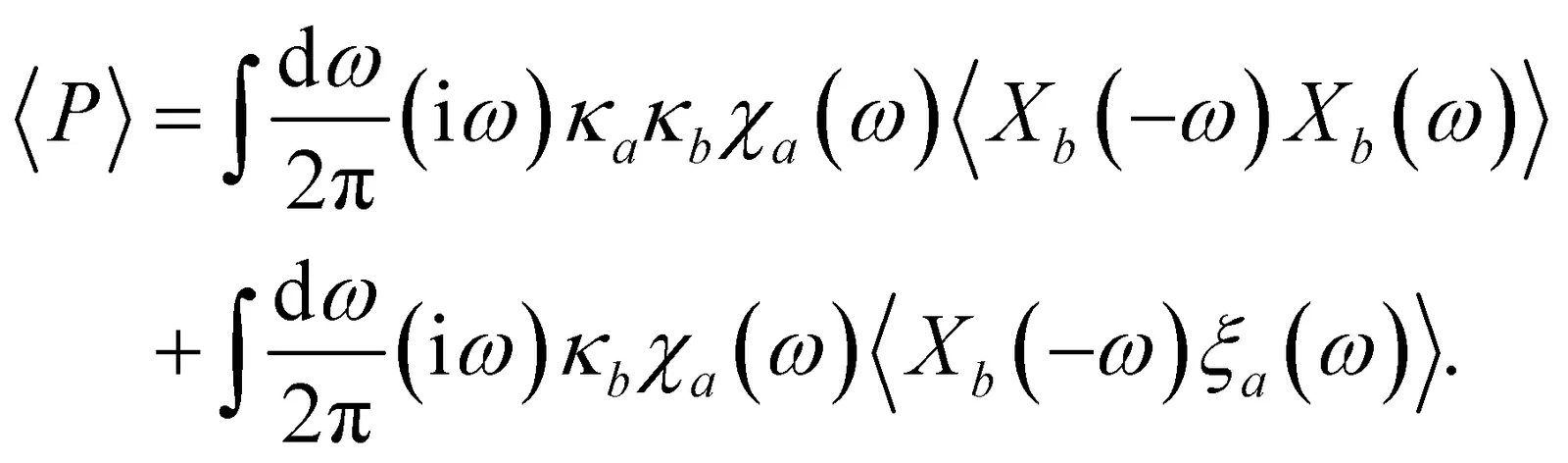


The average power thus separates into two contributions: (i) a term proportional to the autocorrelation 〈*X*_*b*_*X*_*b*_〉, which describes the reactive backaction of the gold membrane and renormalizes the resonance frequency and damping through *Σ*(*ω*) but does not generate a net energy current between baths; and (ii) a term involving the cross-correlation 〈*X*_*b*_*ξ*_*a*_〉, which represents the dissipative contribution arising from thermal fluctuations of the gold bath and governs irreversible energy transfer when *T*_*a*_ ≠ *T*_*b*_.

For a damped harmonic oscillator coupled to a thermal bath at temperature *T*_*j*_, the fluctuation-dissipation theorem gives the force noise spectrumA26
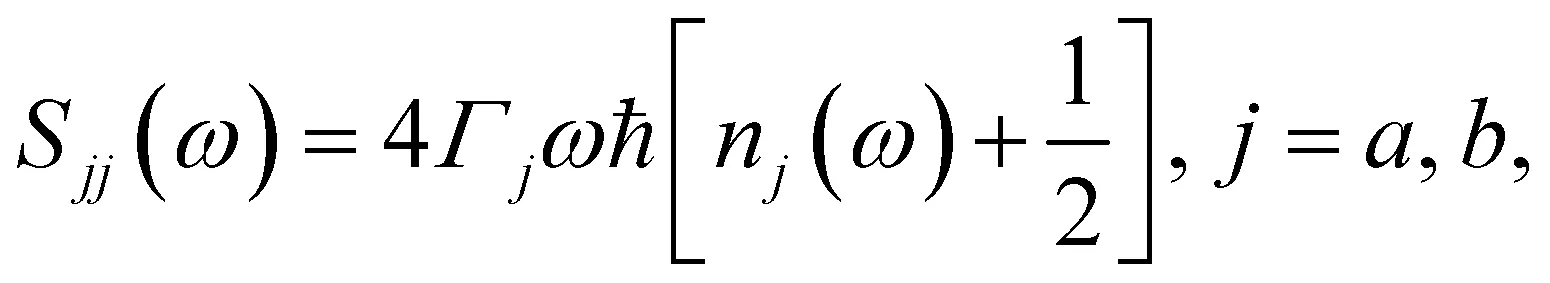
whereA27
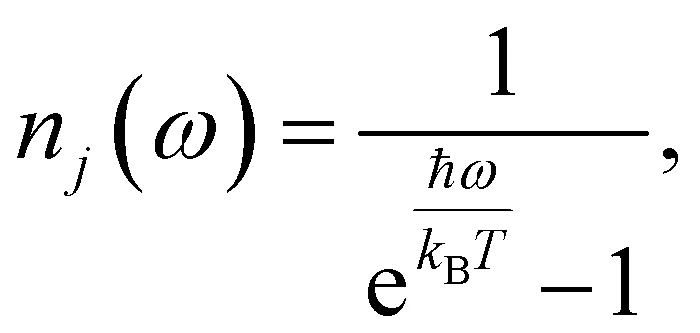
is the Bose–Einstein occupation of bath *j*. The correlator in eqn (A24) can then be evaluated in terms of the graphene Green function *G*_*b*_(*ω*) and the gold susceptibility *χ*_*a*_(*ω*). After straightforward algebra, the net spectral power flowing into the graphene mode can be written in the generic form

A28 *P*_*b*_(*ω*) = 2*ω* Im *Σ*(*ω*)[*S*_*aa*_(*ω*) − *S*_*bb*_(*ω*)],

where *Σ*(*ω*) = *κ*_*a*_*κ*_*b*_*χ*_*a*_(*ω*) is the retarded self-energy introduced above.

This expression has a transparent physical interpretation. The factor Im *Σ*(*ω*), acts as a frequency-dependent spectral transmission function that quantifies how efficiently energy at frequency *ω* is exchanged between the two membranes, while the term in brackets represents the imbalance of fluctuations from the two baths. Since *S*_*jj*_(*ω*) ∝ *n*_*j*_(*ω*), the net power simplifies to

A29 *P*_*b*_(*ω*) ∝ Im *Σ*(*ω*)[*n*_*a*_(*ω*) − *n*_*b*_(*ω*)].

The associated rate of energy flow into the graphene mode can be expressed asA30
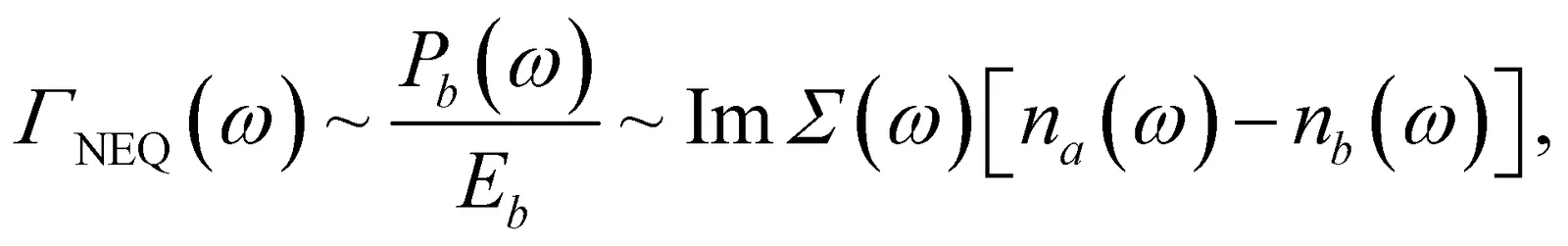
where *E*_*b*_ is the energy stored in the graphene oscillator. Near the graphene flexural resonance, *ω* ≃ *ω*_*b*_, we haveA31
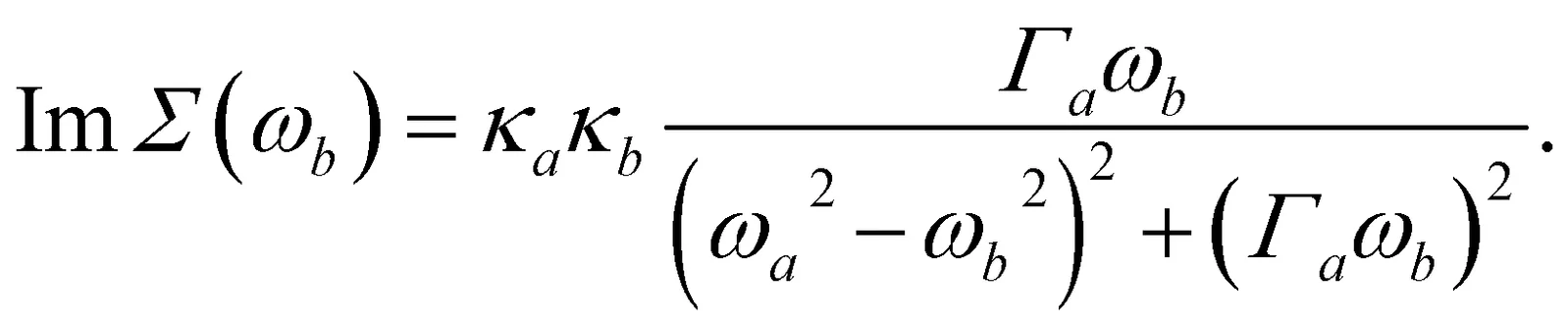


Using the relation
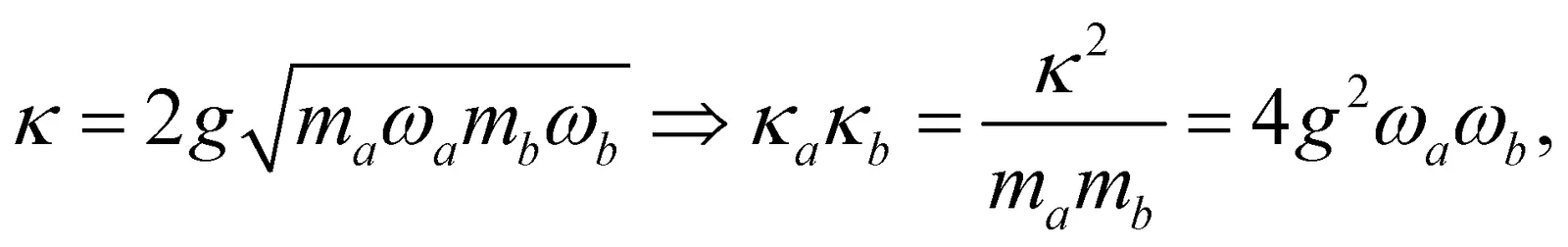
we can rewrite the imaginary part asA32
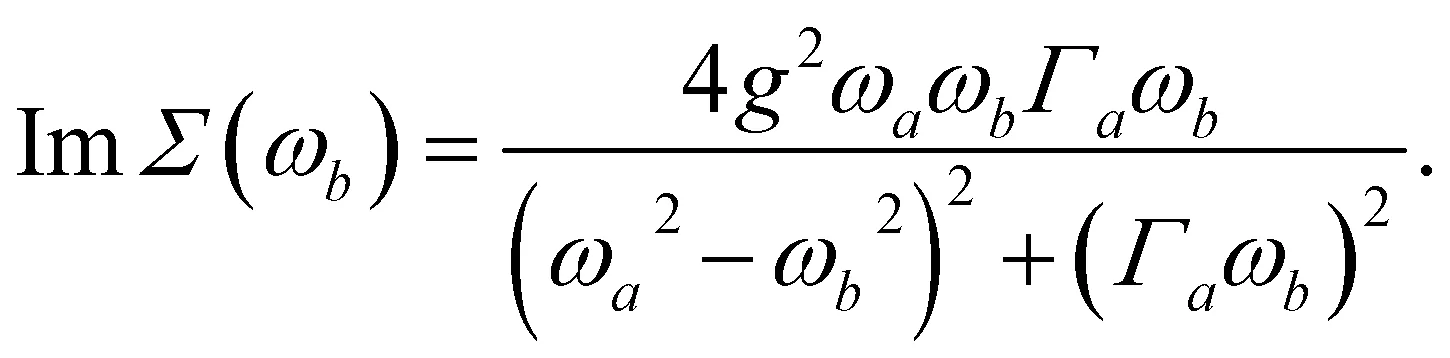


The non-equilibrium pumping rate into the graphene mode at resonance then takes the formA33
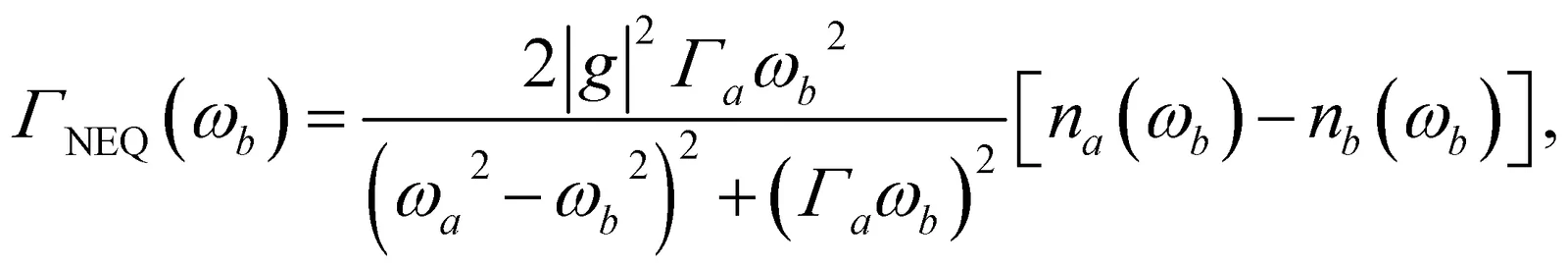
where the numerator sets the maximum pumping rate through the Casimir-mediated coupling and the damping of the gold membrane, the Lorentzian denominator reflects the resonance condition between the gold and graphene modes, and the occupation difference *n*_*a*_ − *n*_*b*_ encodes the non-equilibrium drive. In particular, for *T*_*a*_ > *T*_*b*_, one has *n*_*a*_(*ω*_*b*_) > *n*_*b*_(*ω*_*b*_), so that energy flows from the hotter gold membrane bath into the graphene mode.

It is useful to distinguish the equilibrium contribution to the damping from the additional non-equilibrium pumping contribution. The retarded self-energy generated by the equilibrium Casimir coupling givesA34
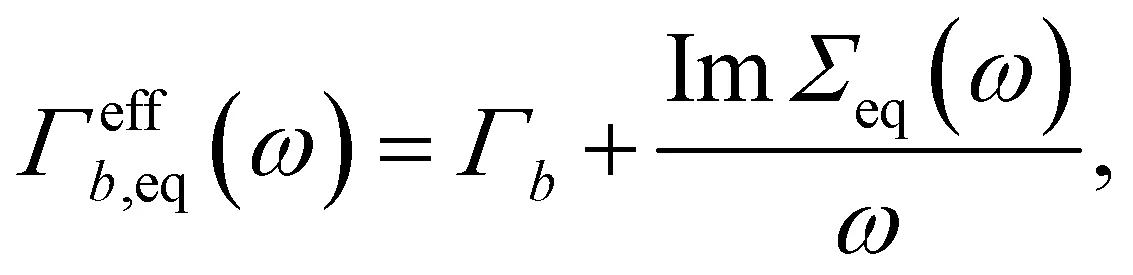
where *Γ*_*b*_ is the intrinsic mechanical damping and Im *Σ*_eq_(*ω*)/*ω* > 0 is the additional equilibrium Casimir-induced damping. This term is positive and therefore does not produce gain.

In the presence of a temperature imbalance, however, the imbalance of fluctuation spectra produces the pumping rate *Γ*_NEQ_(*ω*) derived in eqn (A33). The total effective damping of the graphene mode is thereforeA35
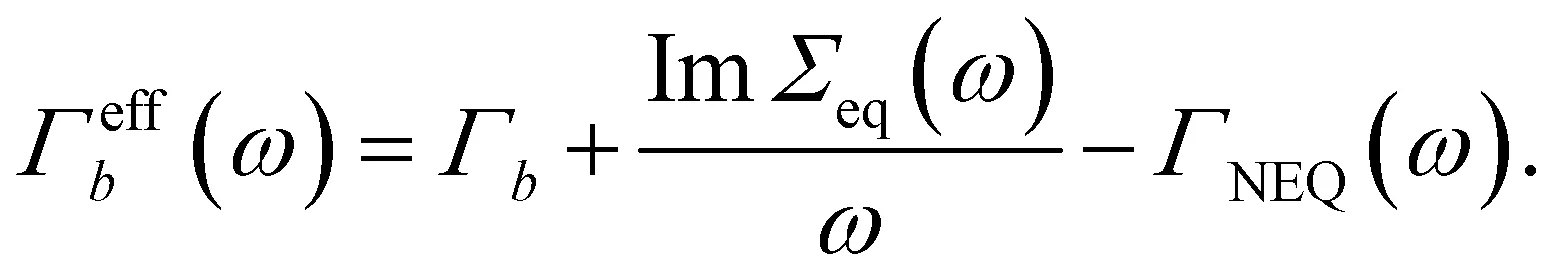


Equivalently, the non-equilibrium pumping contribution may be viewed as a negative imaginary part of the non-equilibrium self-energy,Im *Σ*_NEQ_(*ω*) = Im *Σ*_eq_(*ω*) − *ωΓ*_NEQ_(*ω*).

Thus, *Γ*_NEQ_ does not enter the equilibrium retarded self-energy in eqn (A34); rather, it enters the damping balance through the non-equilibrium fluctuation-induced energy current between the two reservoirs.

For *T*_*a*_ > *T*_*b*_, one has *Γ*_NEQ_(*ω*_*b*_) > 0, and the total effective damping is reduced. If the pumping rate exceeds the sum of the intrinsic and equilibrium Casimir-induced damping channels,A36
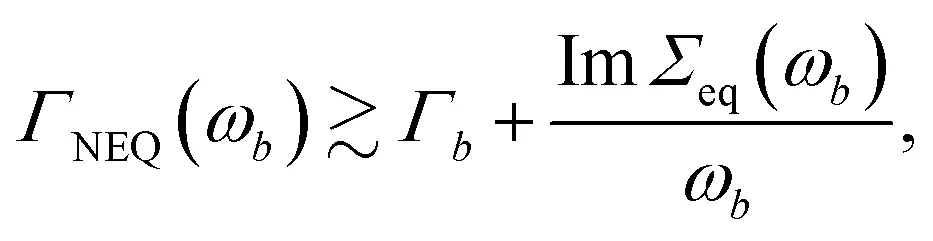
then *Γ*^eff^_*b*_(*ω*_*b*_) < 0, and the graphene flexural mode becomes unstable toward self-sustained oscillations.

Neglecting the small equilibrium broadening term 4*g*^2^/*Γ*_*a*_, the instability threshold reduces toA37
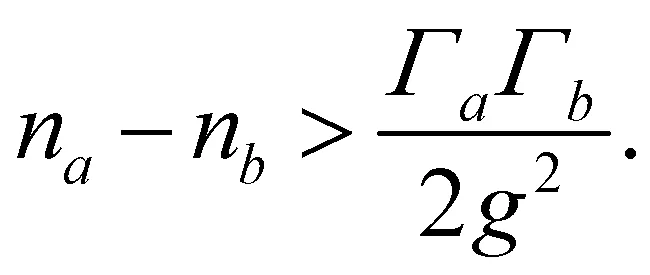


Using the parameters of the graphene and gold membranes considered in this work, the damping rates areA38
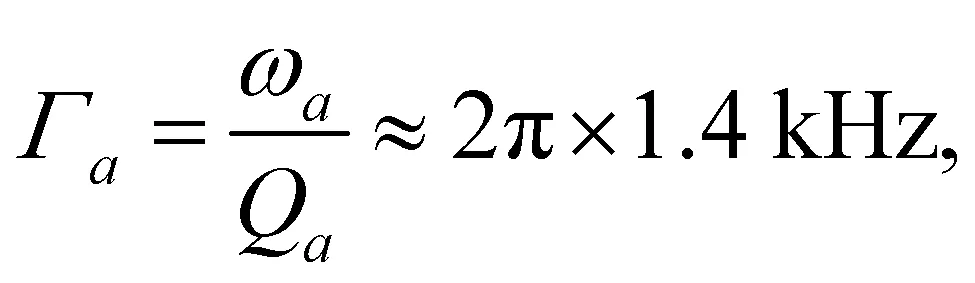
A39
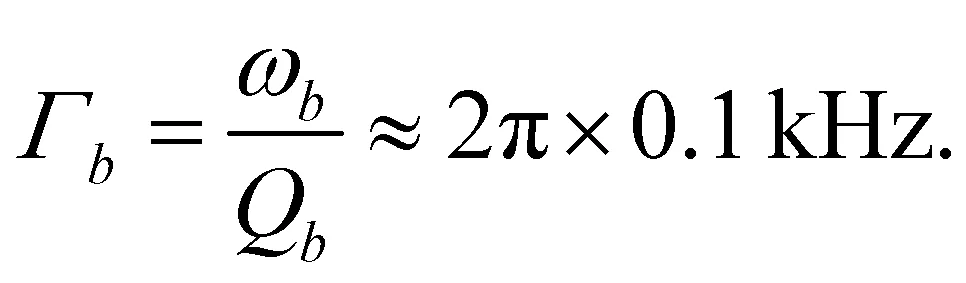


From the above condition, the threshold for non-equilibrium occupation difference isA40
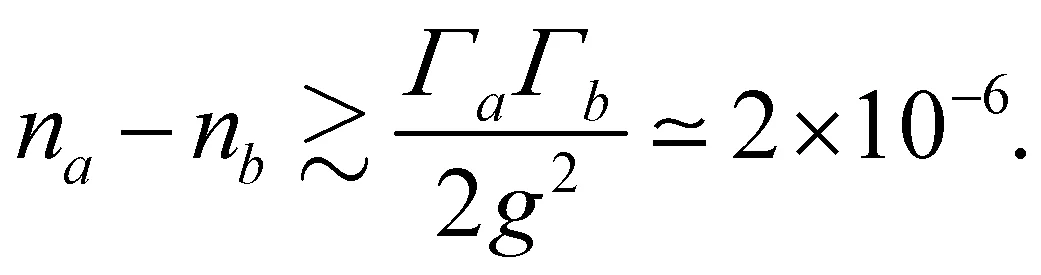


At the experimental temperature *T* = 100 mK, the mechanical occupations are in the classical limit, *n*_*j*_ ≃ *k*_B_*T*/*ℏω*_*j*_ ≫ 1. Using *ℏω*_*b*_/*k*_B_ ≃ 5 × 10^−4^ K, the corresponding temperature difference required to reach threshold is

A41 *T*_*a*_ − *T*_*b*_ ≳ 10^−9^ K.

This extremely small value reflects the simplicity of the resonant, single-mode model. In realistic conditions, additional dissipation channels, imperfect spectral overlap, and mode selectivity increase the required non-equilibrium bias. Nevertheless, this estimate demonstrates that the graphene mode can, in principle, enter the negative-damping regime for modest non-equilibrium conditions.

This non-equilibrium mechanism is distinct from the driven equilibrium hybridization discussed in the main text. In the latter case, the Casimir coupling is static and equilibrium, the imaginary part of the self-energy is positive, and the effective damping remains positive. The observed amplification is thus a driven-response enhancement, not an intrinsic negative damping. By contrast, non-equilibrium Casimir physics can generate true gain. The practical feasibility of such a regime depends on spectral overlap between the fluctuations and the graphene resonance, the inter-membrane separation, the temperature difference, the mechanical quality factors, and possible nonlinearities in the flexural motion or Casimir interaction. These considerations connect to previous proposals where non-equilibrium Casimir forces drive motion and induce energy transfer in nanoscale systems.^83,84^

### 4. Weakly nonlinear saturation of the non-equilibrium instability

The analysis above identifies the linear threshold for the onset of non-equilibrium negative damping in the graphene flexural mode. When the non-equilibrium pumping rate exceeds the sum of intrinsic and equilibrium Casimir-induced damping channels, the effective graphene damping becomes negative,A42
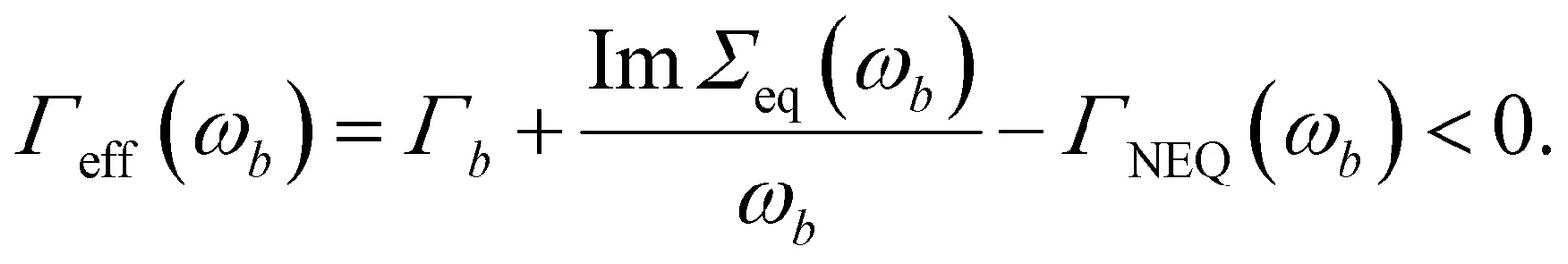


Within the linearized model, this condition implies exponential growth of the graphene displacement amplitude. This growth should not be interpreted as unbounded physical motion. Rather, it signals the breakdown of the linear approximation and the onset of a nonlinear regime in which the amplitude is saturated by membrane nonlinearities, nonlinear dissipation, redistribution of energy among other flexural modes, heating, or, far above threshold, mechanical failure of the suspended structure.

A minimal description of the near-threshold nonlinear dynamics can be obtained by supplementing the effective graphene equation with the leading nonlinear corrections allowed for a suspended membrane.^65,85^ Keeping a Duffing nonlinearity and a phenomenological nonlinear damping term, the real displacement *X*_*b*_(*t*) of the graphene mode obeys, schematically,

A43 *Ẍ*_*b*_ + *Γ*_eff_*Ẋ*_*b*_ + *Ω*_*b*_^2^*X*_*b*_ + *η*_*X*_*X*_*b*_^2^*Ẋ*_*b*_ + *βX*_*b*_^3^ = *ξ*_*b*_(*t*).

here *Ω*_*b*_ is the Casimir-renormalized graphene resonance frequency, *η*_*X*_ is a nonlinear damping coefficient, *β* is the Duffing coefficient, and *ξ*_*b*_(*t*) denotes fluctuations. The Duffing term accounts for the amplitude-dependent frequency shift caused, for example, by geometric nonlinearities and tension renormalization in the suspended membrane.^86,87^ The nonlinear damping term represents the leading dissipative saturation mechanism and is commonly observed in graphene and carbon-based nanomechanical resonators.^65^

Close to the mechanical resonance, it is convenient to introduce a slowly varying complex amplitude *B*(*t*) through

A44 *X*_*b*_(*t*) = *B*(*t*)e^−i*Ω*_*b*_*t*^ + B*(*t*)e^i*Ω*_*b*_*t*^, |*Ḃ*| ≪ *Ω*_*b*_|*B*|.

Substituting eqn (A44) into eqn (A43) and averaging over the fast oscillations at frequency *Ω*_*b*_, or equivalently retaining only the resonant terms in the rotating-wave approximation, gives the standard Stuart–Landau amplitude equation^85^A45
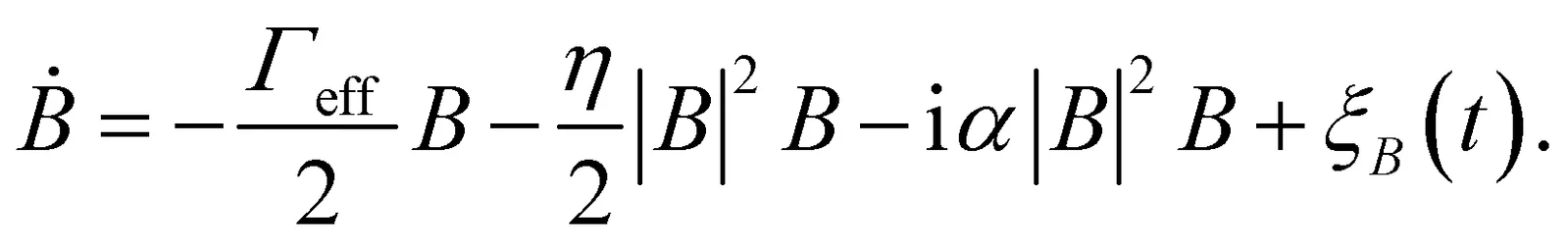


The coefficient *η* > 0 is the effective nonlinear damping coefficient in the slowly varying amplitude description, while *α* is the Duffing-induced amplitude-dependent frequency shift. The term proportional to *α* is purely imaginary and changes the oscillation phase rather than the amplitude. In the present normalization it gives *δΩ*_Duff_ = *α*|*B*|^2^. Equivalently, for a physical peak displacement amplitude *A* = 2|*B*|, the cubic elastic term *βX*_*b*_3 givesA46
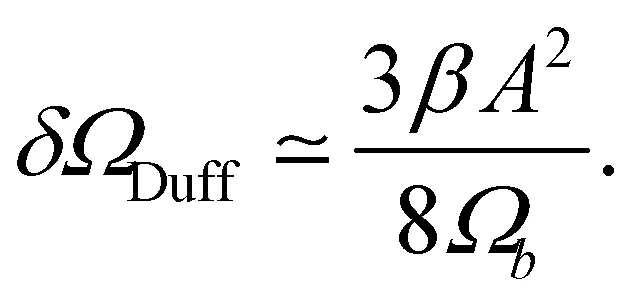


The precise values of *η* and *α* depend on the microscopic nonlinear elastic and dissipative properties of the graphene membrane and on the normalization chosen for *B*. Thus, eqn (A45) should be regarded as a near-threshold normal form rather than as a microscopic derivation of the nonlinear coefficients.

The saturation mechanism is transparent by considering the intensity *I* = |*B*|^2^. Neglecting noise, eqn (A45) gives

A47 *İ* = B*Ḃ + BḂ* = −*Γ*_eff_*I* − *ηI*^2^.

The Duffing term drops out of this equation because it is purely imaginary in the amplitude equation – the term proportional to *α* is purely imaginary in the amplitude equation and, therefore, changes only the oscillation frequency, not the amplitude. The steady-states satisfy

A48 *I*(*Γ*_eff_ + *ηI*) = 0.

For *Γ*_eff_ > 0, the stable solution is when *I* = 0, corresponding to the absence of self-sustained motion in the absence of an external drive. For *Γ*_eff_ < 0, the zero-amplitude state becomes unstable and, provided nonlinear damping is the dominant saturation mechanism, the stable near-threshold amplitude isA49
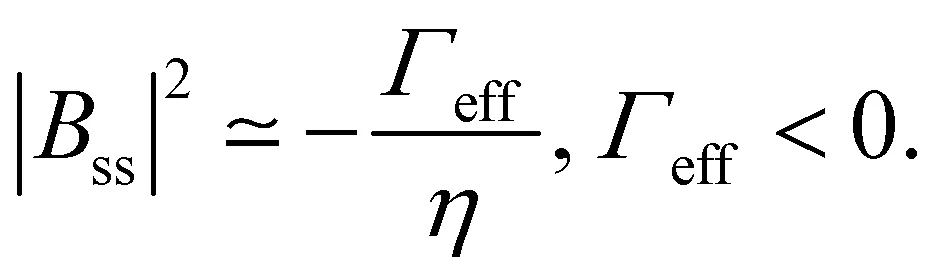


Thus, the negative-damping condition derived in the linear theory should be interpreted as the threshold for the onset of a finite-amplitude self-oscillating state, not as a prediction of indefinite growth.

Although a full microscopic calculation of the nonlinear steady state is outside the scope of the present linear-response framework, one can obtain an order-of-magnitude estimate using known parameters of suspended graphene resonators. The Duffing coefficient of a tensioned or weakly bent graphene membrane is set by geometric nonlinearity and tension renormalization and scales approximately asA50
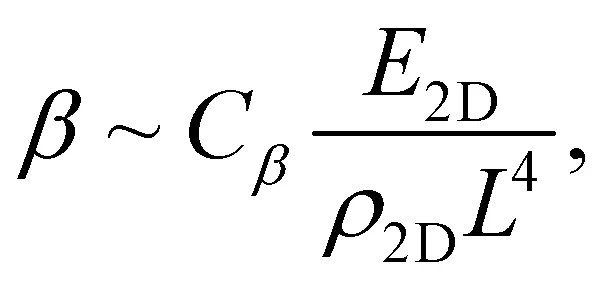
where *E*_2D_ ≃ 340 N m^−1^ is the two-dimensional Young modulus of graphene, *ρ*_2D_ ≃ 7.6 × 10^−7^ kg m^−2^ is its areal mass density,^9^*L* is the lateral membrane size, and *C*_*β*_ is a geometry-dependent factor of order unity. For *L* = 15 µm, this gives *β* ∼ 10^28^–10^29^ s^−2^ m^−2^. From eqn (A46), amplitudes *A* ∼ 1–10 nm at *Ω*_*b*_ ≃ 2π × 13 MHz produce Duffing shifts ranging from tens of Hz to several kHz. These shifts are small compared with the MHz-scale mode spacing but may be observable as amplitude-dependent frequency shifts.

The saturated amplitude is controlled more directly by nonlinear damping. Writing the nonlinear damping coefficient in mass-normalized form as *η*_*X*_ = *η*_nl_/*m*_eff_, with *m*_eff_ ≃ *χρ*_2D_*L*^2^, gives *m*_eff_ ∼ 10^−17^ kg for a 15 µm graphene membrane. Here *χ* is a dimensionless mode-shape factor; for the fundamental mode of a clamped membrane, *χ* is typically of order 0.2–0.3, depending on geometry and boundary conditions. Using experimentally reported nonlinear damping coefficients for graphene and carbon-based nanomechanical resonators,^65^*η*_nl_ ∼ 10^6^–10^8^ kg m^−2^ s^−1^, gives *η*_*X*_ ∼ 10^22^–10^24^ s^−1^ m^−2^. Therefore, for |*Γ*_eff_| ∼ 2π × (0.1–10) kHz, the near-threshold saturated physical amplitude is estimated asA51
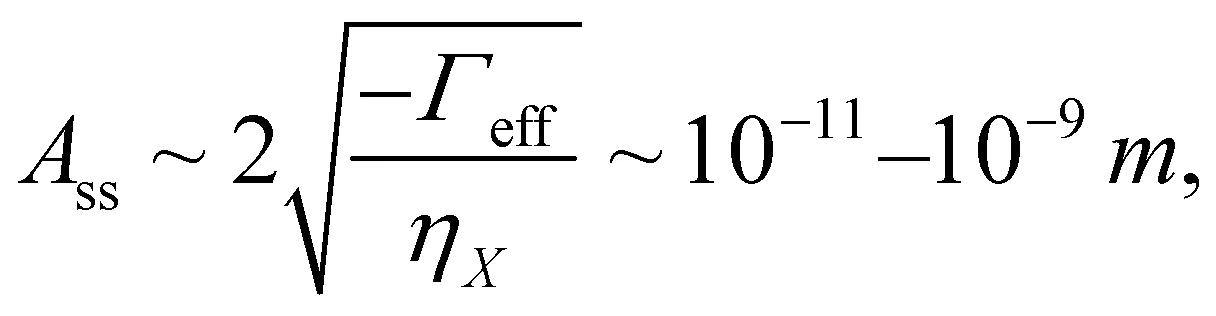
where *A* = 2|*B*| is the peak displacement amplitude. The saturated motion near threshold is therefore expected to remain in the sub-nanometer to nanometer range, consistent with the weakly nonlinear and small-displacement approximations.

This discussion clarifies the scope of the present work. The main results of the manuscript concern the linear spectral signatures of Casimir-mediated hybridization, driven energy transfer, and the threshold for non-equilibrium amplification. A microscopic treatment of the far-above-threshold nonlinear steady state would require determining the Duffing coefficient, nonlinear damping, mode-coupling rates to other flexural modes, and heating-induced changes of the mechanical quality factor for a specific device. These device-dependent effects lie beyond the framework developed here. Nevertheless, the linear threshold, avoided crossings, linewidth modification, and drive-dependent enhancement of the graphene displacement provide experimentally accessible signatures of the Casimir-mediated energy-transfer mechanism.

### 5. Other non-equilibrium routes to negative damping

Beyond temperature gradients, several non-equilibrium configurations can, in principle, produce effective negative damping in Casimir-coupled membranes. These mechanisms rely on driving the system out of equilibrium so that energy flows preferentially into a specific flexural mode.

– Time-dependent modulation of system parameters: if the inter-membrane separation *d*(*t*) or a material property (*e.g.*, dielectric function or conductivity) is modulated at frequencies near twice the mechanical resonance, the Casimir interaction becomes explicitly time dependent. Such modulation can parametrically pump the flexural mode, leading to negative effective damping and self-sustained oscillations. This scenario is fundamentally different from the scheme considered in the present work. Here, the Casimir coupling is static, and the gold membrane is driven externally. While energy is transferred coherently to the graphene membrane *via* hybridization, Im *Σ*(*ω*) remains positive and the graphene damping does not become negative. Accordingly, the observed classical amplification is a driven response enhancement, not true negative damping.

– Relative motion between membranes (quantum friction): a small relative velocity between the membranes induces non-equilibrium fluctuations and quantum friction. Fluctuating electromagnetic fields can transfer energy preferentially into the flexural mode of one membrane, effectively reducing its damping. This mechanism has been theoretically proposed as a way to generate motion from vacuum fluctuations in sliding nanosystems.^42^

– Externally pumped electromagnetic environment: placing the membranes in a cavity or near a driven electromagnetic field that selectively excites vacuum fluctuations at the mechanical resonance can create an effective non-equilibrium bath. In this case, the graphene mode can absorb energy from the environment without direct mechanical driving, potentially producing negative damping and self-oscillation.

– Asymmetric material dissipation: if one membrane has internal dissipation mechanisms that preferentially convert vacuum fluctuations into coherent motion in the graphene mode, the effective damping can be reduced. Nonlinearities in the flexural dynamics or in the Casimir coupling can enhance this effect.

In summary, non-equilibrium Casimir forces can, in principle, produce genuine negative damping and self-sustained motion; these scenarios require either time-dependent modulation, relative motion, or engineered non-equilibrium baths. The driven equilibrium hybridization in our study enhances the graphene response but does not produce intrinsic negative damping.

## Data Availability

The data that support the findings of this article are openly available.^82^
